# The sertraline metabolite, desmethylsertraline, may be implicated in adverse outcomes reported after gestational sertraline use: insights from a study in zebrafish

**DOI:** 10.1007/s43440-025-00765-y

**Published:** 2025-07-28

**Authors:** Cassius M. Phogole, Lesha Pretorius, Tracy Kellermann, Maré Vlok, Carine Smith

**Affiliations:** 1 Division of Clinical Pharmacology, Department of Medicine, Faculty of Medicine and Health Sciences, Stellenbosch University, Cape Town, South Africa; 2 Experimental Medicine Group, Department of Medicine, Faculty of Medicine and Health Sciences, Stellenbosch University, Cape Town, South Africa; 3 Central Analytical Facility: Mass Spectrometry, Faculty of Medicine and Health Sciences, Stellenbosch University, Cape Town, South Africa

**Keywords:** SSRI, Proteomics, Larvae, Cardiovascular risk, Locomotion, SERT, Equivalent dose

## Abstract

**Background:**

Sertraline (SER) is a selective serotonin reuptake inhibitor (SSRI) prescribed for depression, including during pregnancy. Existing literature suggests a potential association between gestational SER use and cardiac and neurodevelopmental anomalies in exposed offspring. This study evaluated the relative impacts of SER and its metabolite desmethylsertraline (DES) on the proteome during early development.

**Methods:**

Zebrafish embryos and larvae were exposed to individual treatments of translated umbilical cord-blood equivalent concentrations of SER or DES during early developmental stages. Quantified activity tracking and protein expression levels of serotonin transporter (SERT) were used to confirm a significant SSRI effect in exposed larvae. A whole-larvae proteomic analysis was conducted using a label-free quantitative liquid chromatography-mass spectrometry approach. Protein identification was performed using zebrafish and human protein databases.

**Results:**

Apparent therapeutic SSRI effect of exposure doses of SER and DES was confirmed in zebrafish larvae, by reduced activity levels, as well as decreased SERT. DES, but not SER, resulted in several differentially regulated proteins, identified in both the zebrafish and human databases. The results from the two databases correlated and aligned with an increased risk for cardiovascular and neurodevelopmental dysregulation.

**Conclusion:**

Proteomic data suggest that DES, rather than SER, at physiologically relevant doses, may be responsible for adverse clinical outcomes reported after gestational SSRI use. Current data positions larval zebrafish as a possible tool for assessment of long-term risk after gestational SER use, as well as a drug development tool in this context.

**Clinical trial number:**

N/A.

**Supplementary Information:**

The online version contains supplementary material available at 10.1007/s43440-025-00765-y.

## Introduction

Depression, a common psychological disorder, is a debilitating, chronic or recurrent illness that influences the socio-economic functioning of patients [1, 2]. It is also prevalent during pregnancy, affecting more than 10% of pregnant women worldwide [3]. Given the severity of untreated gestational depression, which can lead to maternal suicidal behaviour, reduced self-care, non-adherence to prenatal guidelines, and impulsivity that may jeopardize the health of both mother and child [4–7], selective serotonin reuptake inhibitors (SSRIs) are commonly prescribed for its management. Among these, sertraline (SER) is frequently prescribed due to its relatively favourable safety profile [8–13]. In the context of pregnancy, SSRIs, specifically SER and its major active metabolite, desmethylsertraline (DES), are known to cross the placental barrier [14, 15]. Notably, DES generally exhibits blood concentrations that are two-fold higher than those of its parent drug, SER, including in umbilical cord blood [16–18], which raises concerns. Moreover, emerging literature suggests a potential association between SSRI use during pregnancy and an increased risk of cardiac and neurodevelopmental anomalies in exposed offspring [14, 15, 19, 20]. Despite these detrimental health concerns, the mechanisms by which SSRIs, particularly SER, impact foetal development remain poorly understood.

In the early development of neural circuits, serotonin (5-HT) plays a pivotal role in cell division, differentiation, and migration, as well as neuron myelination, dendritic pruning, and synaptogenesis, prior to its well-known role in the mature brain as a neurotransmitter modulating cognition, emotions, pain, feeding, sleep, and arousal [19, 21, 22]. Consequently, SSRI-induced dysregulation of 5-HT synthesis in the developing brain could potentially have a long-term effect on the foetal neural networks [19, 22]. This dysregulation may also impact other 5-HT signalling pathways. For example, preclinical studies suggest a critical role of 5-HT in cardiac morphogenesis [23–25]. In these studies, embryos exposed to elevated concentrations of 5-HT exhibited reduced proliferation of myocardial, mesenchymal, and endothelial cells. In the developed human heart, 5-HT regulates cardiac contractile function via 5-HT_4_ receptors [26]. Therefore, dysregulation of 5-HT during development may also increase the risk of congenital heart defects.

Therefore, the present study evaluated the impact of prolonged exposure to SER and DES at translated cord blood-equivalent concentrations in larval zebrafish during early development. Experimental endpoints included quantification of activity levels and protein expression levels of the serotonin transporter (SERT) – which is targeted by SSRI – to allow interpretation regarding the physiological relevance of exposure doses used. Additionally, whole proteome analysis of exposed larvae was conducted to provide a holistic view of potentially affected pathways.

## Materials and methods

### Animal husbandry and ethical compliance

All animal-based procedures were conducted in accordance with the South African National Standard guidelines for care and use of animals in research. All experimental protocols in wild-type zebrafish (*Danio rerio*) were ethically cleared by the Stellenbosch University Research Ethics Committee for Animal Care and Use (ref #ACU-2021-21995). The zebrafish eggs were produced in the Zebrafish Husbandry Unit within the Department of Medicine at Stellenbosch University using standardized husbandry methods and provided within 4 h post-fertilisation (hpf). Zebrafish eggs and larvae were maintained under standardized husbandry conditions, in E3 (embryo medium) prepared as previously described [27].

To reduce the number of live organisms used in this investigation, data for all experimental groups were simultaneously generated, so that only one control group was required. However, for clarity of results presentation, we present the effects of SER and DES separately (but compared to the same controls).

### Treatment dose determination and administration

No standard formula for the conversion of human doses to equivalent doses for use in zebrafish currently exists. However, we have previously developed a formula for the dose conversion of anxiolytic substances known to act via modulation of serotonergic signalling – we demonstrated the calculated equivalent doses to have a significant therapeutic effect in a larval zebrafish model of anxiety [28], which supports the accuracy of our calculations. We thus employed the same formula in the current study, calculating a human equivalent dose (HED) for zebrafish from interquartile ranges of actual human umbilical cord blood concentrations of SER and DES reported in mothers treated with SER during pregnancy [16]. This formula accounts for extracellular fluid and blood volumes, effectively treating zebrafish as a body tissue. Additionally, in our context, the increased extracellular fluid and blood volumes during pregnancy were considered.

The initial stock solutions of SER (Sertraline hydrochloride; 98% purity, Toronto Research Chemicals (TRC); cat no: 4-DAR-9-1) and DES (*rac*-cis-N-desmethylsertraline hydrochloride; 97% purity, TRC; cat no: 1-MOZ-80-1) were prepared at a concentration of 1 mg/mL in dimethylsulfoxide (DMSO). Serial dilutions were then performed with E3 medium to achieve final concentrations of 10, 25, and 50 ng/mL for SER and 15, 20, and 60 ng/mL for DES, with negligible final levels (< 1%) of DMSO [29]. These were considered to be the absolute bioavailabilities at the tissue level in zebrafish, considering the fact that zebrafish readily absorb treatments through both gills and skin when exposed to treatments via immersion [30]. The treatment groups were maintained in E3 medium containing either SER or DES from 4 to 118 hpf, while the control group was maintained in E3 medium (i.e. no treatment additives) only. Media were refreshed every 24 h to replenish treatments and remove waste products. The stability of the analytes in E3 medium for 24 h was not assessed, as a previous study demonstrated the stability of SER in E3 medium for up to 5 days [31].

In all experiments, larvae were assessed daily for any obvious signs of drug toxicity, such as pericardial oedema and spinal curvature [32], using a stereomicroscope. Mortality and hatching rate were also recorded. Furthermore, to facilitate visualisation after immunofluorescent labelling of SERT, 75µM 1-phenyl 2-thiourea (PTU) was added to the E3 media of larvae earmarked for microscopy, for the duration of the drug administration protocol, to limit the development of skin pigmentation.

### Experimental endpoints

The experimental endpoints, assessed at 118 hpf, included behavioural evaluation (*n* = 72 per group, achieved by three separate experiments) using the light-dark transition test (LDTT), following a standard protocol described elsewhere [28]. Immunofluorescence staining and subsequent quantification of protein expression levels of serotonin transporter (SERT, *n* = 10 per group) were conducted in the larval brain. Additionally, whole proteome analysis (*n* = 55 pooled larvae per sample, with 3 separate samples analysed per experimental group) was performed using a label-free quantitative liquid chromatography-mass spectrometry (LC-MS) approach, following exposure to selected concentrations of 25 ng/mL for SER and 20 ng/mL for DES. Proteomic database interrogation was performed using the individual UniProt databases (http://www.uniprot.org) for *Danio rerio* (zebrafish) and *Homo sapiens* (humans), as zebrafish exhibit conserved vertebrate biology and share about 70% genetic similarity with humans [33, 34]. For all experiments except the proteomic analysis, euthanasia was conducted via overdose with MS-222, followed by freezing at -20 °C. For the proteomic analysis, the euthanasia procedure involved snap-freezing in liquid nitrogen before protein extraction for analysis. Detailed protocols for the behavioral (activity) assay, whole-mount immunohistochemistry (SERT), and proteomic analysis are provided in the supplementary materials.

### Statistical analyses

Data is presented separately for SER and DES to improve clarity of graphs, but both were compared to the same control group. GraphPad Prism (version 9.4.0; CA, USA) was used for statistical analyses and graphical visualization of data from all experiments, except for the proteomic analysis, where the built-in statistical analysis in FragPipe Analyst was applied (refer to supplementary material for detailed description of this process). Outlier identification was performed in all groups using the ROUT method (Q = 1%) to exclude deviating values before calculating mean values. The normality of the data was assessed using the D’Agostino & Pearson test. For parametric data (activity data), group comparisons were conducted using a one-way ANOVA and Tukey’s post-hoc tests, with results reported as means ± SD. In addition, behavioural data were also binned into equal time intervals (1-minute bins) for visualization and qualitative assessment. This data is presented as means ± SEM. For normally distributed data with significantly different variances around the mean (SERT), the Brown-Forsythe ANOVA and Dunnett’s T3 multiple comparisons test were used, and results reported as means ± SD.

## Results

### SER and its metabolite, DES, exhibited reduced locomotory activity in the LDTT

**Fig. 1:**
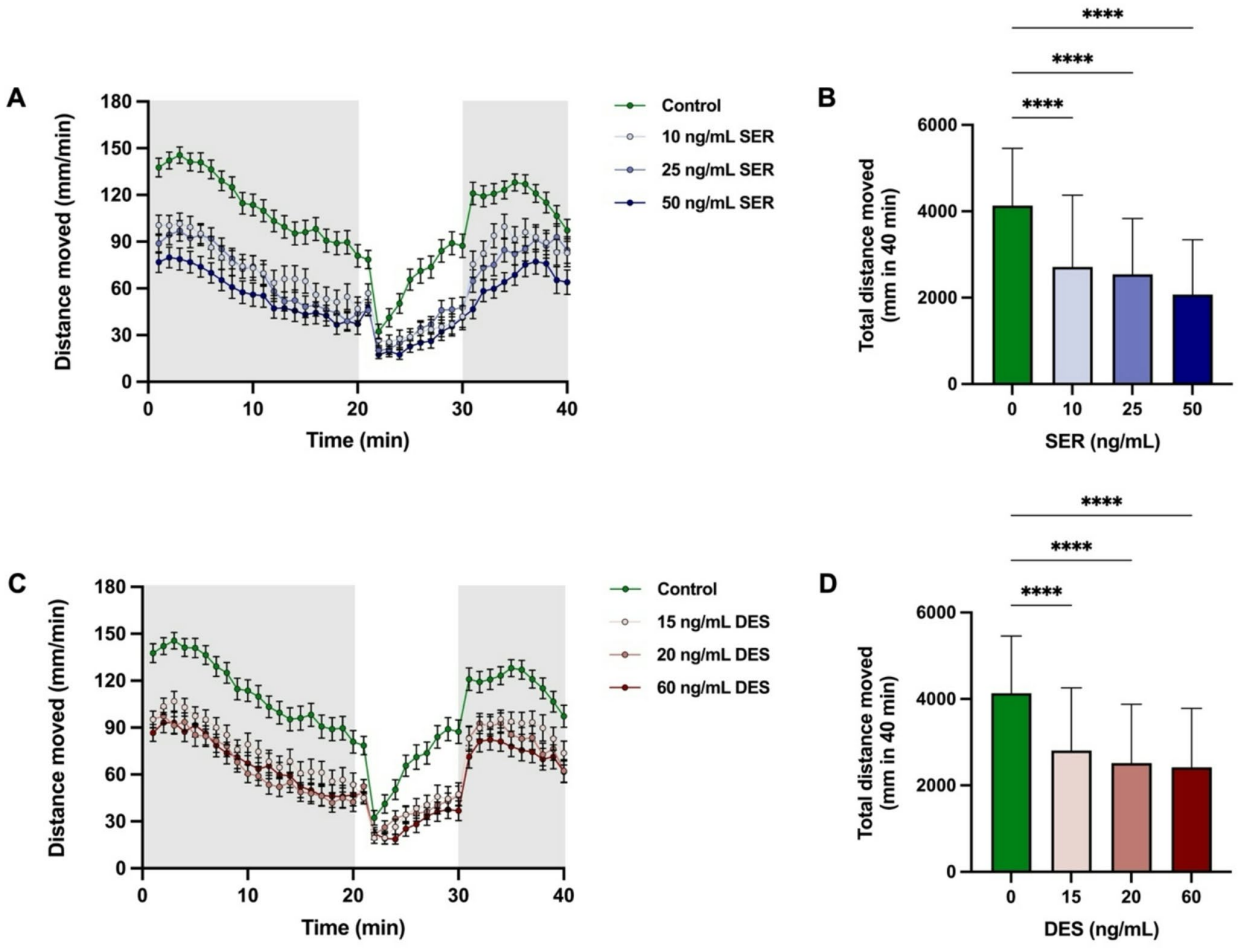
Locomotor activity following prolonged exposure (from 4 to 120 h post fertilisation) to sertraline (SER, **A** & **B**) and desmethylsertraline (DES, **C** & **D**) at concentrations equivalent to cord blood levels. The activity tracks (**A** & **C**) demonstrate changes in movement during a light-dark transition test (LDTT). Tracks are binned into 1-min intervals, and data are represented as mean ± SEM. The total distance moved throughout the LDTT (**B** & **D**) is also displayed. Data are represented as mean ± SD, and statistical analysis was performed using a one-way ANOVA test with post-hoc Tukey’s test to indicate differences between groups with ****, *p* < 0.0001. These analyses were performed on 3 different batches of larvae, total *n* = 60–70 per group

Although small decreases were observed in 24 h survival in both the SER and DES treated groups vs. control, as well as a transiently delayed hatching at 48 h in most treated groups (supplementary material; Figure S1), no signs of overt toxicity was evident, which is in line with previously reported data on SER exposure at similar doses [35]. Figure 1 illustrates the behavioural responses of zebrafish larvae after sequential exposure to bright white light and darkness in the LDTT, following SER and DES administration during their early developmental phase. Although the magnitude of the responses was generally similar between both the treated and control groups, which all exhibited normal LDTT profiles (Fig. 1A, C), the treated groups exhibited significantly lower overall activity levels (Fig. 1B, D; SER, F_3,246_ = 26.93; DES, F_3,245_ = 22.26). These findings align with other studies reporting decreased locomotion in larval zebrafish following SSRI exposure [35, 36] and suggest that the human equivalent concentrations used indeed achieved therapeutic levels in the larvae. Of further importance, although both SER and DES groups exhibited relative hypolocomotion, the general profile of the response to the light-dark transition was not altered (arguing against sedation or overt toxicity as reasons for decreased activity).

### Changes in serotonin transporter level after exposure to SER and DES

**Fig. 2:**
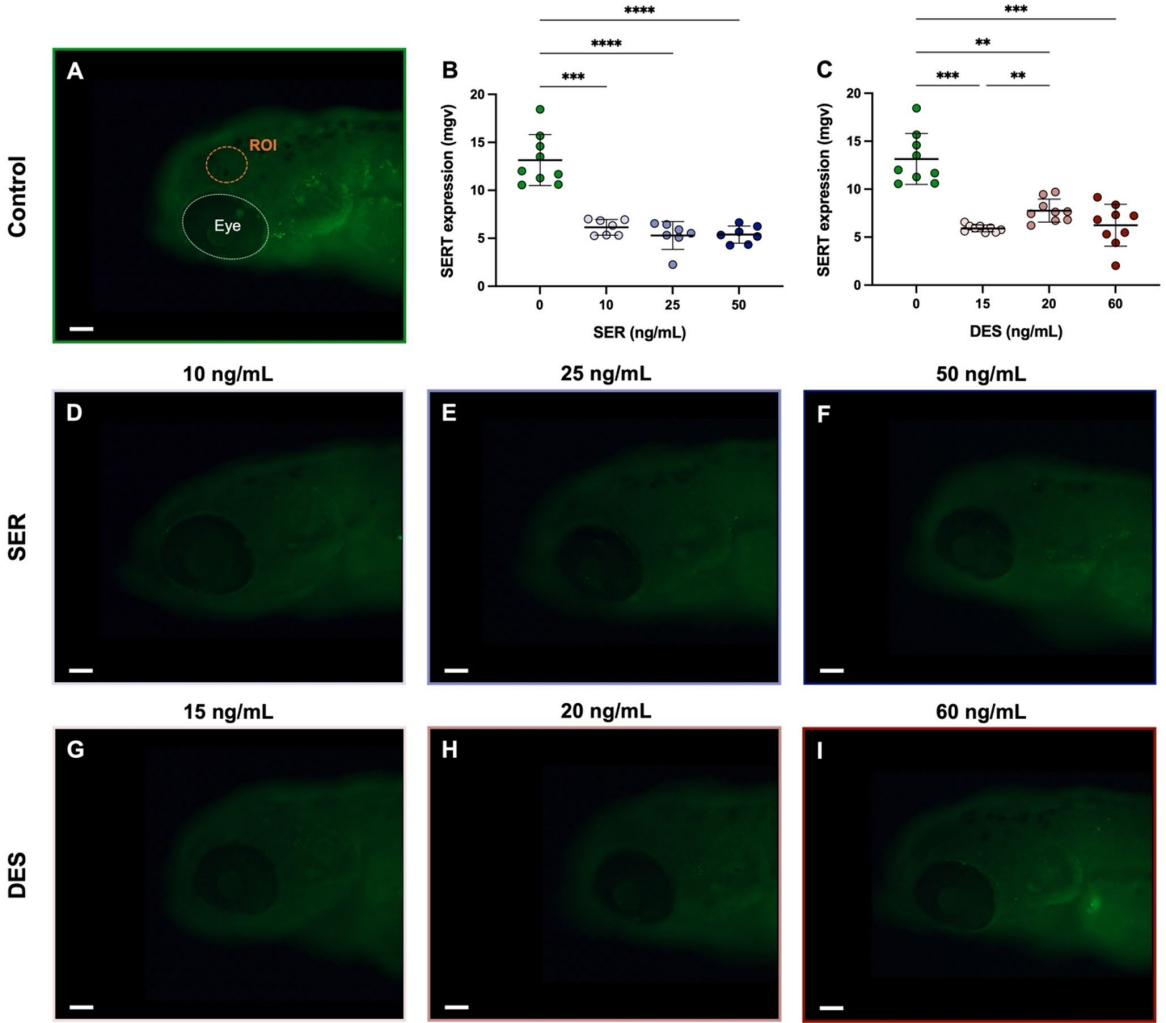
Serotonin transporter (SERT) expression levels following prolonged exposure (from 4 to 120 h post fertilisation) to sertraline (SER) and desmethylsertraline (DES) at concentrations equivalent to cord blood levels. The region of interest (ROI) in the larval head indicates the area quantified upon image analysis (**A**). In addition, the larval eye is indicated as a reference point. Quantified mean grey values (mgv) are represented for SER (**B**) and DES (**C**), respectively. These data are represented as individual data points with mean ± SD, *n* = 7–9 per group. Statistical analyses was performed using a Brown-Forsythe ANOVA test with Dunnett’s T3 multiple comparisons test to indicate differences between groups with **, *p* < 0.01; ***, *p* < 0.001; and ****, *p* < 0.0001. Representative fluorescent images of SERT expression for control (**A**), SER (**D**-**F**), and DES (**G**-**I**) are depicted. Larvae were imaged using a 10x objective lens at 100x magnification; scale bar = 50 μm

Immunohistochemistry analysis for the expression of SERT supported our interpretation of the behavioural data and further confirmed that a therapeutic level antidepressive-like effect was achieved in the current study. Exposure to all tested doses of both SER and DES resulted in significant downregulation of SERT expression (Fig. 2; SER, F_3,16_ = 46.94; DES, F_3,19_ = 30.24). Additional representative images may be viewed in the supplementary material (Figure S2).

### DES, rather than its parent drug, SER, affected the proteome profiles of zebrafish larvae

**Fig. 3:**
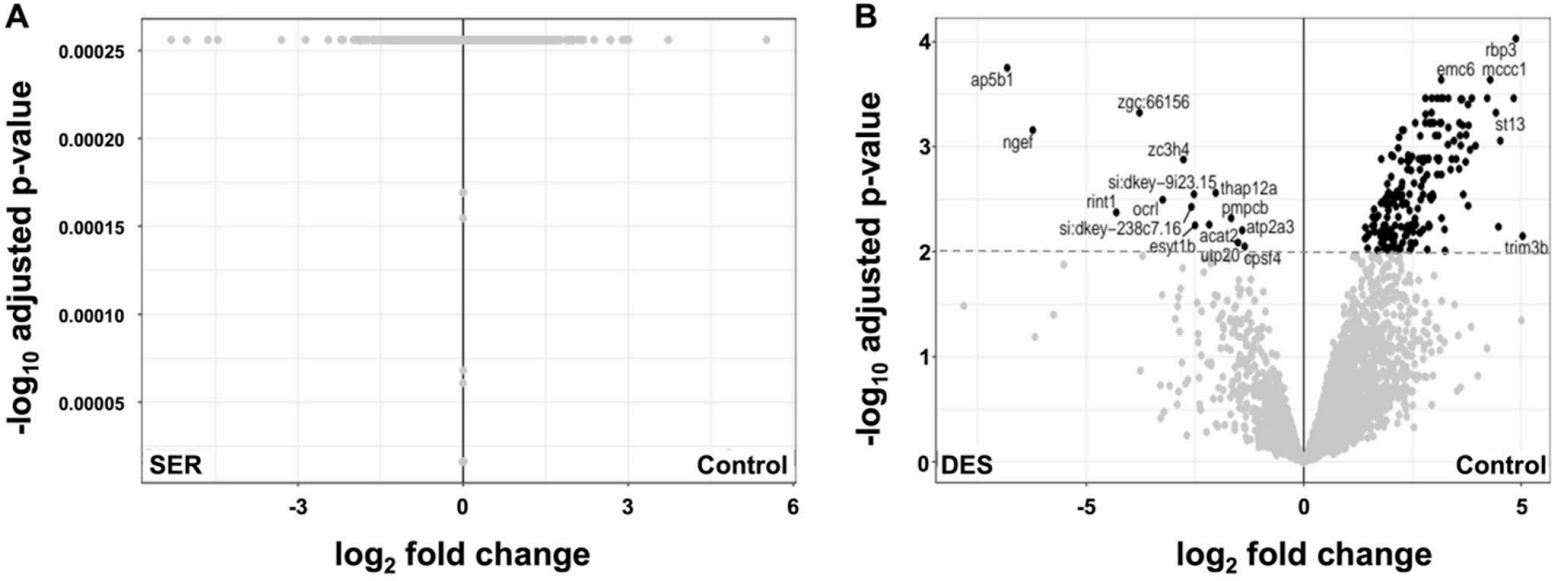
Volcano plots illustrating the comparative analysis of zebrafish larval proteomes using the zebrafish database for protein interrogation following chronic exposure (114 h) to sertraline (SER; 25 ng/mL) (**A**) and desmethylsertraline (DES; 20 ng/mL) (**B**) relative to a control group, to identify differentially regulated proteins. A cutoff of an adjusted p-value of 0.01 along with a log2-fold change of 1 was applied to determine significantly regulated proteins in each pairwise comparison. The labelled proteins above the cutoff in Figure B represent those that were significantly downregulated

The proteome of whole zebrafish larvae was analysed using a label-free quantitative LC-MS approach to better understand the effects of early exposure to SER (25 ng/mL) and DES (20 ng/mL) during developmental stages, particularly concerning neurodevelopmental and heart defects in exposed offspring. Data processing and analysis were performed using the MSFragger workflow and FragPipe Analyst in data-independent acquisition (DIA) mode (Figure S3). Protein identification was conducted using independent human and zebrafish databases (Figure S4). The zebrafish database yielded 3 046 reproducibly identified proteins (Table S1,S2), whereas interrogation with the human database resulted in 616 identified proteins (Table S3). Notably, no differentially regulated proteins were observed for the SER-exposed vs. control group in either the human or zebrafish databases (Figs. 3A and 6A). Consequently, no further analysis was conducted for this group. However, several differences were observed in larvae exposed to DES.

When considering the data generated from the zebrafish database first, 188 out of 3 046 reproducibly identified proteins were significantly upregulated, whereas 15 out of 3 046 were significantly downregulated (Fig. 3B).

**Fig. 4:**
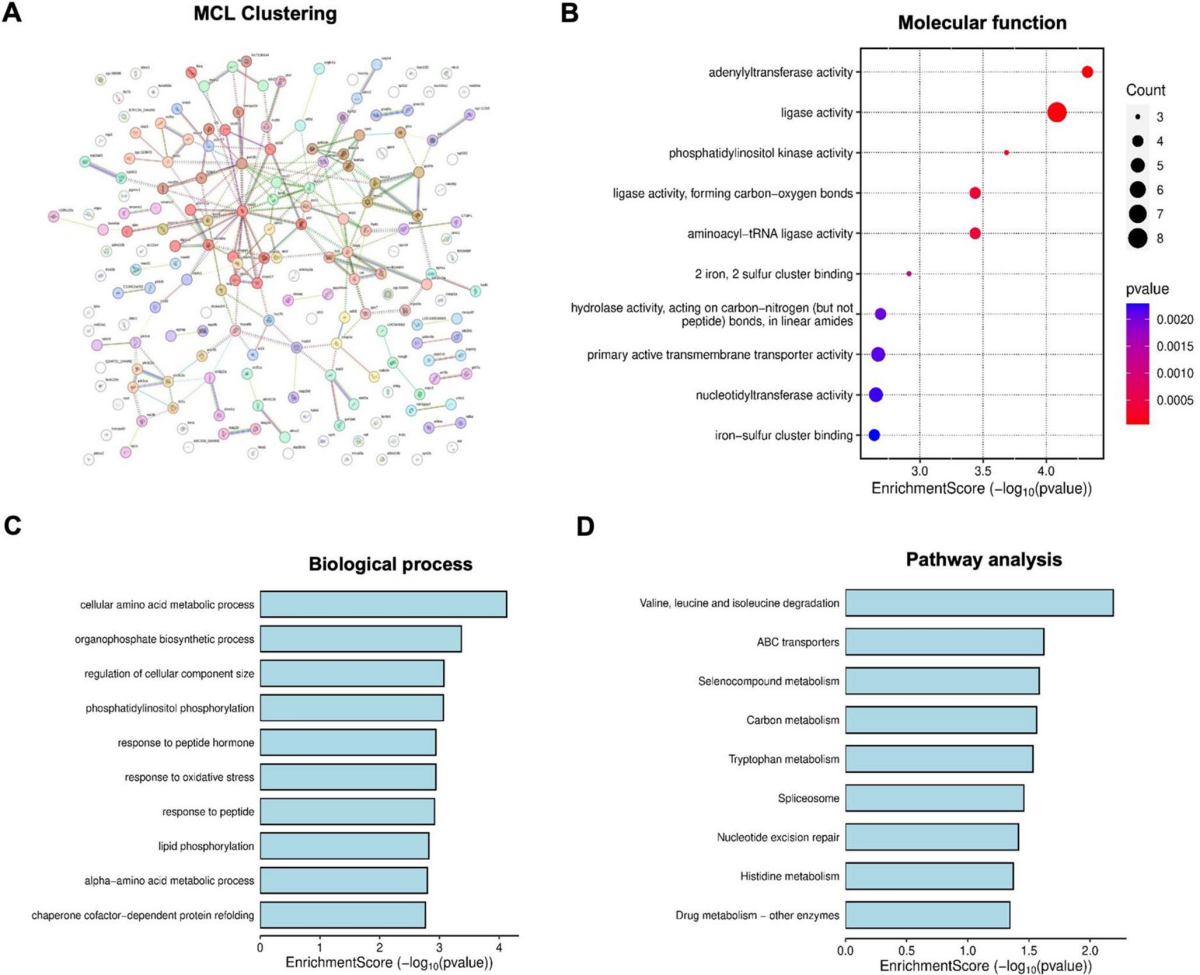
Markov Cluster Algorithm (MCL) clustering results from the STRING database and GO (Gene Ontology) enrichment analysis of upregulated proteins (interrogated using a zebrafish database) in zebrafish larvae treated with desmethylsertraline (DES; 20 ng/mL) for 114 h (refer to Table S1 for specific proteins). Protein-protein interaction (PPI) regulatory network (**A**): Nodes represent proteins, and edges denote protein-protein associations. Green, pink, and black lines indicate text-mining, experimentally determined interactions, and co-expression, respectively. Refer to supplementary material for identification of all identified differentially regulated proteins. Molecular functions (**B**), biological processes (**C**), and pathway analysis (**D**) were identified through GO enrichment analysis. The x-axis represents the -log10 of p-values or enrichment scores, while the y-axis displays up to 10 identified significant items, ranked in increasing order of significance (refer to Figure S5 for higher-resolution images)

**Fig. 5:**
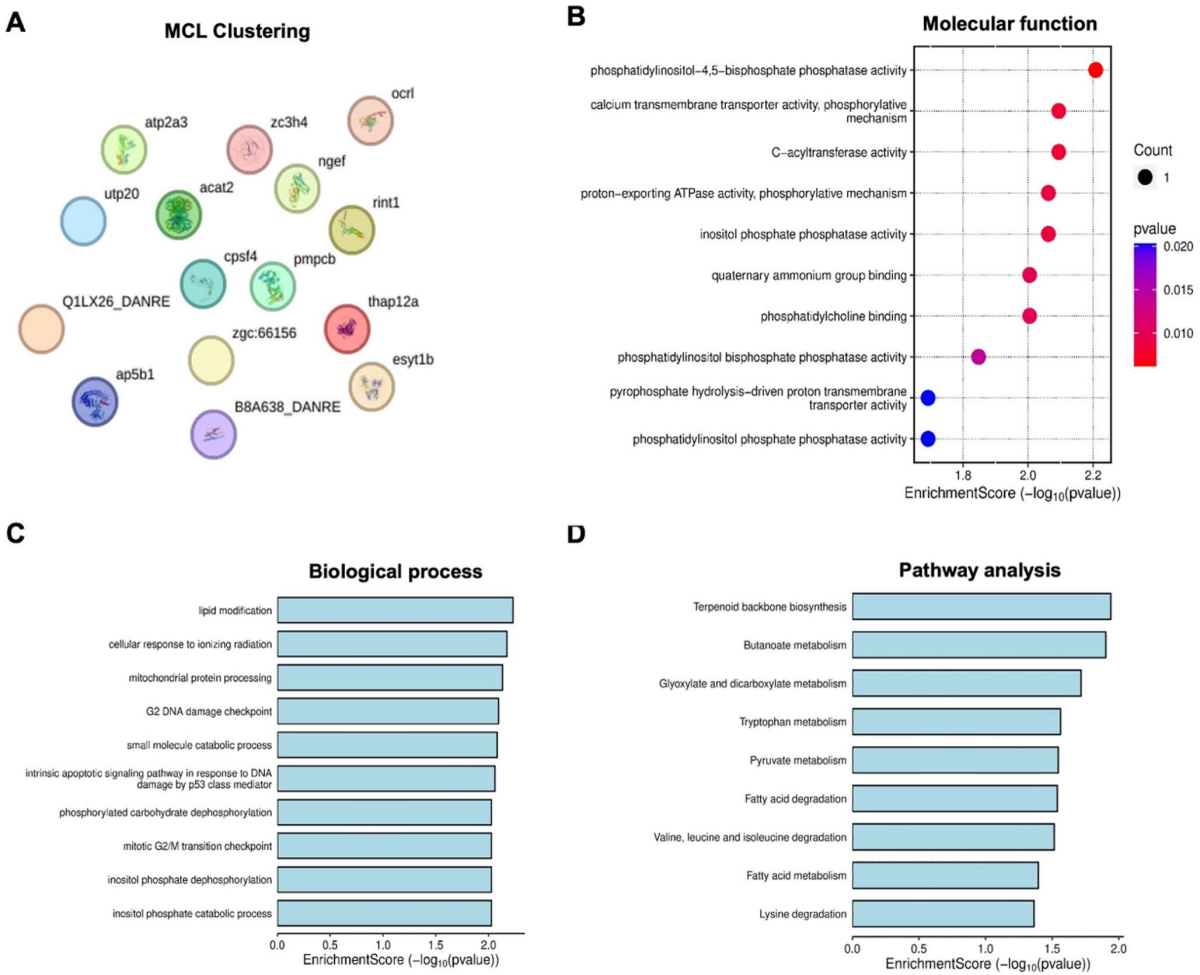
Markov Cluster Algorithm (MCL) clustering results from the STRING database and GO (Gene Ontology) enrichment analysis of downregulated proteins (interrogated using a zebrafish database) in zebrafish larvae treated with desmethylsertraline (DES; 20 ng/mL) for 114 h (refer to Table S2 for specific proteins). Protein-protein interaction (PPI) regulatory network (**A**): Nodes represent proteins, and edges denote protein-protein associations. Green, pink, and black lines indicate text-mining, experimentally determined interactions, and co-expression, respectively. Refer to supplementary material for identification of all identified differentially regulated proteins. Molecular functions (**B**), biological processes (**C**), and pathway analysis (**D**) were identified through GO enrichment analysis. The x-axis represents the log10 of p-values or enrichment scores, while the y-axis displays up to 10 identified significant items, ranked in increasing order of significance (refer to Figure S6 for higher-resolution images)

The protein-protein interactions (PPIs) among the up- and downregulated proteins identified in the DES-treated groups were constructed using the STRING database (version 12.0) to better understand their associations and roles in the biological system. For upregulated proteins, 47 PPIs were identified (Fig. 4A). Of these, the two main interaction networks involved mitochondrial translation elongation and riboflavin metabolism, both of which play key roles in energy production. The mitochondrial translation elongation PPIs included 13 nodes (TBCA, RPL19, SEC61g, PKLR, SLTM, MRPL2, NME2A, PTCD3, MRPS17, MRPL45, DIP2CA, NDUFA9A, and SLC27A1A) and 24 edges (Fig. 4A, S5A), with an enrichment p-value < 0.0001. The PPIs involved in riboflavin metabolism included 8 nodes (PUS1, ACP1, FLAD1, H6PD, CAT, SDR16C5A, WUFB14D09, and ILVBL) and 12 edges, with an enrichment p-value < 0.0001. No PPIs were identified for downregulated proteins using the zebrafish database (Fig. 5A).

In addition, a Gene Ontology (GO) analysis was conducted using the Bioinformatics website (http://www.bioinformatics.com.cn/*).* This analysis of dysregulated proteins using the zebrafish database identified several implicated molecular functions, biological processes and pathways (Figs. 4 and 5). These included tryptophan metabolism and various energy metabolic processes, such as pyruvate metabolism, fatty acid degradation, carbon metabolism and branched-chain amino acid (valine, leucine, and isoleucine) degradation. Additionally, the ABC transporters pathway, which regulates the influx and efflux of substances across cell membranes, was significantly enriched.

**Fig. 6:**
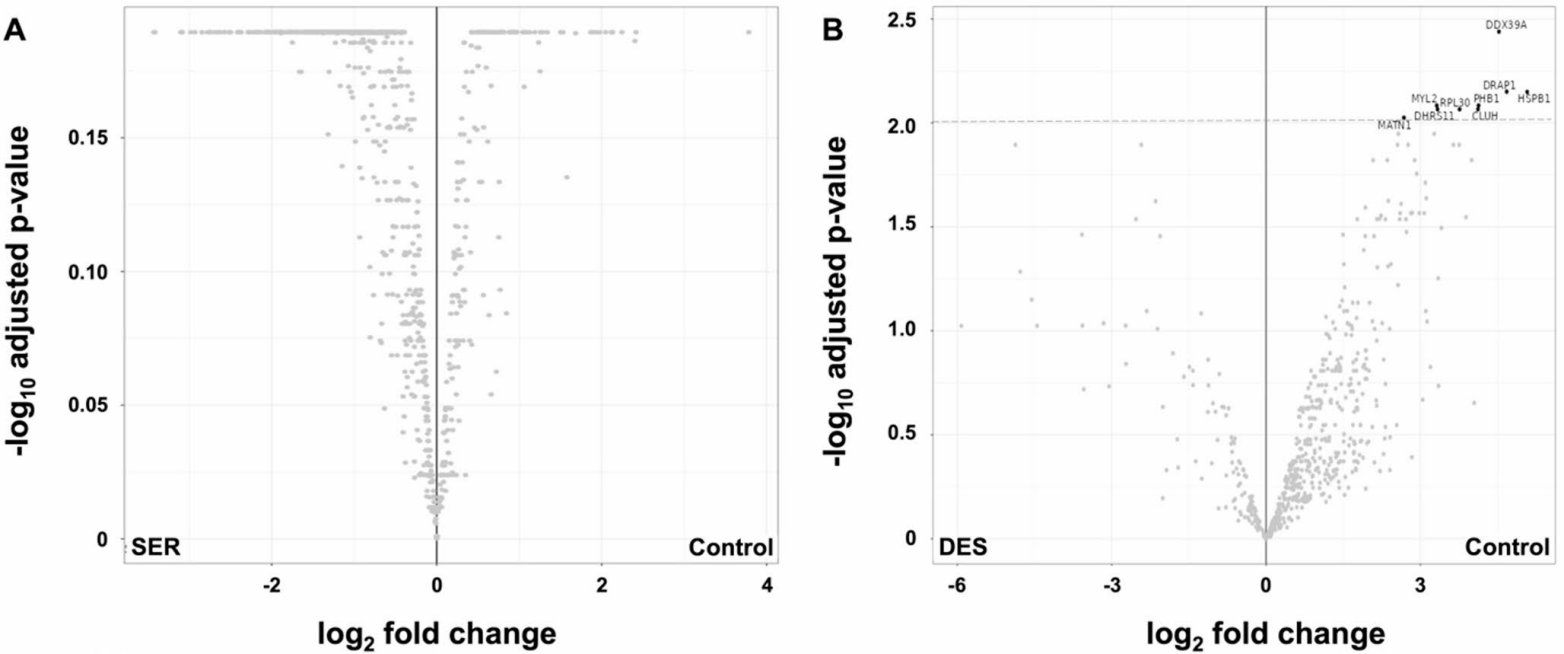
Volcano plots illustrating the comparative analysis of zebrafish larval proteomes using a human database for protein interrogation following chronic exposure (114 h) to sertraline (SER; 25 ng/mL) (**A**) and desmethylsertraline (DES; 20 ng/mL) (**B**) relative to a control group, to identify differentially regulated proteins. A cutoff of an adjusted p-value of 0.01 along with a log2-fold change of 1 was applied to determine significantly regulated proteins in each pairwise comparison. The labelled proteins above the cutoff in Figure B represent those that were significantly downregulated

Turning attention to proteins identified using the human database, in the DES-exposed group, 9 out of 616 reproducibly quantified proteins were significantly downregulated when compared to the control group (Fig. 6B).

**Fig. 7:**
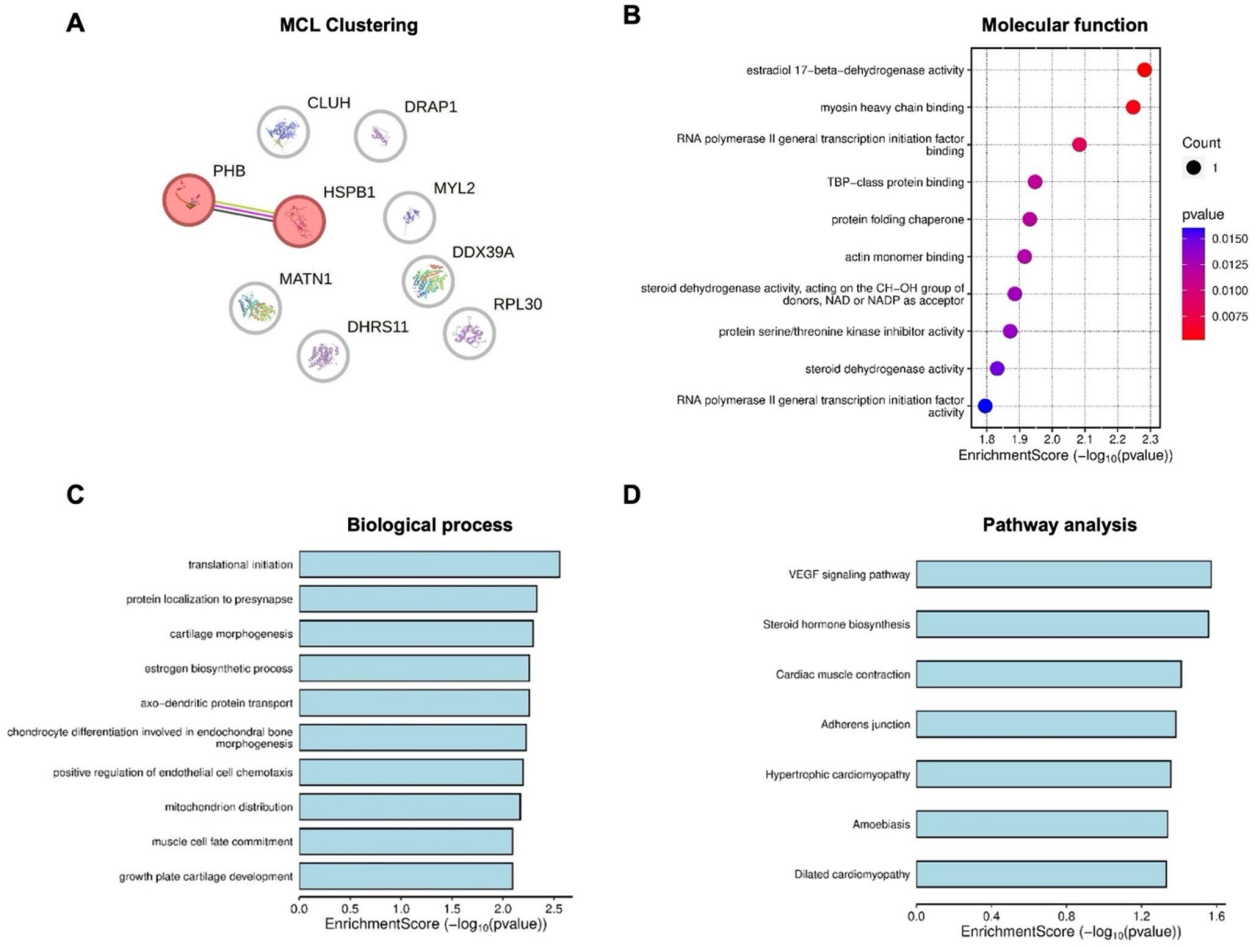
Markov Cluster Algorithm (MCL) clustering results from the STRING database and GO (Gene Ontology) enrichment analysis of downregulated proteins (interrogated using a human database) in zebrafish larvae treated with desmethylsertraline (DES; 20 ng/mL) for 114 h (refer to Table S3 for specific proteins). Protein-protein interaction (PPI) regulatory network (**A**): Nodes represent proteins, and edges denote protein-protein associations. Green, pink, and black lines indicate text-mining, experimentally determined interactions, and co-expression, respectively. Molecular functions (**B**), biological processes (**C**), and pathway analysis (**D**) were identified through GO enrichment analysis. The x-axis represents the log10 of p-values or enrichment scores, while the y-axis displays up to 10 identified significant items, ranked in increasing order of significance (refer to Figure S7 for higher-resolution images)

The protein-protein interactions (PPIs) analysis based on the human database revealed only two proteins, HSPB1 and PHB1, which exhibited interaction networks (Fig. 7A), with two nodes and one edge. The PPI enrichment p-value was 0.055. HSPB1 is known to play a role in stress resistance and actin organization, while PHB1 is involved in inhibiting DNA synthesis and regulating cell proliferation.

GO enrichment analysis of downregulated proteins using the human database (Fig. 7) identified several molecular functions, including a rate-limiting enzyme in steroid biosynthesis, estradiol 17-beta-dehydrogenase activity, and a key protein involved in muscle contraction, specifically the myosin heavy chain (MHC) binding protein. Most of the highlighted pathways are related to the cardiovascular system, such as the vascular endothelial growth factor (VEGF) pathway, which is a master regulator of angiogenesis [37, 38]. Additionally, other significantly enriched pathways included steroid hormone biosynthesis and adherens junctions.

## Discussion

The effects of SER exposure on zebrafish larvae at environmentally relevant concentrations have been studied in terms of behaviour and selected molecular systems [35, 36, 39, 40]. However, to the best of our knowledge, this is the first study to report the effects of prolonged exposure to physiologically relevant concentrations of SER and DES in zebrafish larvae on the serotonergic system, behavioural outcomes, and proteomic profiles. Using equivalent doses of SER (25 ng/mL) and DES (20 ng/mL) – derived from actual human umbilical cord blood concentrations – we demonstrated firstly that these doses achieved SSRI-associated effects (decreased SERT protein expression levels and relative hypolocomotion) in zebrafish larvae, and secondly, that exposure of larvae to these “therapeutic” doses of DES in particular, resulted in proteomic dysregulation during early developmental stages that aligns with comorbidity risk in human offspring after *in utero* exposure to these compounds.

In the current study, the generally lower motility observed under both unstimulated and stimulated conditions, in zebrafish larvae treated with SER and DES, aligns with other studies of SSRI exposure in larval zebrafish [35, 36]. While the cited studies – which had somewhat different aims to ours (e.g. environmental toxicity of SER) – assessed effects of SER at administration doses of up to 100 ug/l, it is important to note that in the current study, the SSRI-like effect on locomotion was observed at (equivalent) doses previously reported in human umbilical cord blood [16]. In addition, the current study expands on available literature by confirming that even after metabolism into DES, this effect is maintained.

This interpretation is supported by the data on SERT protein expression. SERT plays a crucial role in regulating 5-HT levels, and SSRIs exert their antidepressant effects by inhibiting SERT-dependent serotonin reuptake into presynaptic neurons, resulting in increased synaptic 5-HT levels and prolonged neurotransmission [41, 42]. In terms of quantification of SERT levels in zebrafish, variable findings have been reported for messenger ribonucleic acid (mRNA) expression levels of SERT in larvae exposed to SER (6-144 hpf), with lower concentrations (1 and 10 ng/mL) showing elevated SERT levels vs. a higher concentration (100 ng/mL) showing unaltered levels [39]. Furthermore, both a clinical study [43] and a study in rats [44] previously reported reduced SERT levels, but not SERT genotype (mRNA) per se, to be associated with affective disorders such as elevated anxiety. Building upon these findings, in the current study, we opted for quantification of SERT protein expression levels rather than mRNA levels. Current data showed that SERT protein levels were significantly inhibited in both SER and DES exposed larvae, thus supporting an interpretation of physiologically relevant SSRI effects of both SER and DES in this model.

Practically, in terms of longer-term health outcome, achieving SSRI-effect during early developmental stages via SER or DES transfer across the placenta, may lead to a heightened risk of relative 5-HT toxicity in offspring exposed to SER and DES during early development. Indeed, in the clinical setting, excessive 5-HT activity has been associated with a range of symptoms, including neurological disturbances such as anxiety and confusion, autonomic dysfunction like arrhythmias, and neuromuscular abnormalities [45, 46]. Impairment of the serotonergic system during early developmental stages may have a long lasting effects, as both low and high 5-HT exposure during the critical perinatal period have been linked to behavioural deficits in adulthood [47]. Current study design did not allow for evaluation of potential counter-adaptations in terms of 5-HT receptor function. Available literature in this context have focused on mRNA levels and/or gene expression only, rather than protein levels or signalling capacity of 5-HT receptors. Thus, the interplay of receptors other than SERT (as reported in the current study), remains to be elucidated.

For example, limited previous data have suggested increased SER signalling via 5-HT_2B_ to influence cardiovascular development, also suggesting that zebrafish is a relevant model for studying 5-HT and its interaction with its receptors [48]. In the context of the current study, these data align with cardiovascular risk reported in offspring exposed to SER *in utero*. This warrants further studies investigating the effects of SER exposure during early development, in terms of modulated sensitivity of 5-HT-receptors (5-HT_2B_ and others).

The proteomic analysis of larvae following exposure to SER (25 ng/mL) and DES (20 ng/mL) revealed significant effects of DES – but not SER itself – on the larval proteome. Given the expected relatively high abundance of proteins with a developmental role in zebrafish larvae (which may pose a limitation to proteome analysis and interpretation), as well as the high homology of zebrafish proteins to those of humans, a parallel protein identification were performed using both zebrafish- and human-protein databases. With this approach, a more comprehensive understanding of DES-associated dysregulation at protein level was achieved.

Firstly, from the zebrafish protein database, upregulated proteins were mainly linked to mitochondrial translation elongation and riboflavin metabolism, both of which are vital for optimal mitochondrial ATP production across cell types. Of specific relevance to the current topic though, the heart has been named as primary target of the mitochondrial translation elongation factor (TUFM) gene dysfunction [49], suggesting that the heart may be particularly vulnerable in scenarios where these proteins are dysregulated. In addition, dysregulated processes implicated (pyruvate metabolism, fatty acid degradation, carbon metabolism and branched-chain amino acid degradation), as well as the ABC transporters pathway, suggest that metabolic processes dependent on mitochondrial ATP production, were already compromised in the larvae. These observations align with the higher risk for cardiovascular disease in offspring after gestational SER use and provides some insight into possible mechanisms at play.

The prominence of downregulation of proteins implicated in tryptophan metabolism is another significant concern, as this may further exacerbate mitochondrial function [50]. Furthermore, altered levels of tryptophan, SERT and mitochondrial dysfunction has been linked to several neurodevelopmental and psychiatric disorders [9, 14, 15, 19, 50].

Turning attention to insights from protein identification using a human protein database, in the DES-treated group, the downregulated proteins (DDX39A, CLUH, HSPB1, MYL2, MATN1, PHB1, RPL30, DRAP1, and DHRS11) identified are primarily involved in key biological pathways, showing high correlation with pathways identified from the zebrafish protein database. In addition to those related to the cardiovascular system and mitochondrial functions, proteins implicated in steroid biosynthesis and cellular adherens junctions were also identified. Together, these data expand on potential dysregulated mechanisms elucidated from the zebrafish protein database.

For example, the identified pathways related to the cardiovascular system included the VEGF signalling pathway, cardiac muscle contraction, as well as dilated and hypertrophic cardiomyopathies. The low expression of these proteins, particularly MYL2 (which plays a key role in heart development and function [51]) highlights the increased risk of cardiovascular complications. Indeed, common congenital adversities reported in offspring exposed to SSRIs during pregnancy include heart defects [20, 52–54]. The downregulation of MYL2 may result from altered 5-HT levels during early development, as observed in response to SSRI exposure, since 5-HT is known to play a role in cardiac signalling pathways [23–26, 55]. Steroid hormone biosynthesis was another identified pathway via the estrogen biosynthesis process. This process appears to be facilitated by the downregulated DHRS11 protein, which plays a key role in steroid biosynthesis, including estrogen production [56]. Estrogen is primarily involved in the female reproductive system, regulating sexual organ differentiation as well as the development and maintenance of the reproductive system [57]. Additionally, this sex hormone exhibits pleiotropic effects on other systems, including the nervous, cardiovascular, immune, and musculoskeletal systems [57]. Estrogen deficiency has been linked to serious medical conditions such as osteoporosis, arthralgia, cardiovascular disease, Alzheimer’s disease, and Parkinson’s disease [58]. This may be an explanation for high incidences of cardiovascular and bone diseases in postmenopausal women, where estrogen (particularly estradiol) is produced at lower levels [59, 60]. Thus, these findings may indicate sex bias, with female offspring exposed to DES potentially at higher risk when compared to male offspring.

The adherens junction was another identified pathway modulated by proteins such as DRAP1 and MATN1, both of which were found to be downregulated. Adherens junctions play critical roles in initiating and stabilizing cell-cell adhesion, mediating intracellular signalling, and regulating gene transcription as well as the actin cytoskeleton [61]. DRAP1 is known to induce functional repression of both activated and basal transcription of class II genes [62], while MATN1, which binds to collagen, plays a significant role in the extracellular matrix of non-articular cartilage [63]. Consequently, the biological processes identified included cartilage morphogenesis, chondrocyte differentiation involved in endochondral bone morphogenesis, and growth plate cartilage development. A previous study has highlighted musculoskeletal malformations as a type of congenital defect in offspring exposed to SSRIs during pregnancy [20].

Notably, the STRING database searches identified a direct interaction between downregulated proteins PHB1 and HSPB1, potentially via shared mitochondrial functions and maintenance. PHB1 is a key regulator of autophagic degradation of mitochondria [64, 65], whereas HSPB1 plays a role in cytoskeletal elements, including preventing cytoskeletal damage, protecting cells from various stressors, and exerting anti-apoptotic effects at multiple levels [66]. Given the essential role of mitochondria in maintaining crucial metabolic pathways, regulating the oxidative stress response, apoptosis, and generating cellular energy [65, 67], disruption of this organelle could impact many biological processes. Indeed, mitochondrial dysfunction has been implicated in various pathophysiologies, including cardiovascular diseases such as atherosclerosis, cardiomyopathy, and myocardial infarction, as well as autism spectrum disorders (ASD) [65, 68].

Biological process analysis identified the translational initiation process, possibly mediated by downregulated proteins DDX39A and/or RPL30. DDX39A is involved in pre-mRNA splicing and is essential for mRNA export from the nucleus [69], whereas RPL30 plays a crucial role in protein synthesis [70]. Although the exact function of RPL30 remains unclear, a preclinical study in rodent models suggests that it may play a role in neurulation and contribute to the risk of neural tube defects [71]. This could explain the increased risk of neural tube defects reported in the offspring of mothers who used SSRIs during the first trimester [20, 52, 53, 72].

Taken together, dysregulated proteins identified from both human and zebrafish databases, aligns in terms of organ systems at risk. In our opinion, the use of both databases for protein identification is a good practice and maximises information yield from a single proteome extraction – while the zebrafish database seem to provide a larger number of identified proteins, those identified in the human database may be more easily related to data from clinical studies. Thus, validation of larval zebrafish drug exposure as an accurate disease risk model may be more accurately determined using a human protein database, while the zebrafish-specific protein identification may provide more leads in terms of mechanisms involved, or therapeutic target identification. Nevertheless, the fact that both databases returned a picture of dysregulation of the proteome – which aligns with comorbidities which manifest clinically only later in life - at very early developmental stages already, suggest that early remedial intervention should be a priority.

In summary, the close alignment of current findings to increased risk for comorbidities described in children from mothers using SER during pregnancy, validates the zebrafish as model to investigate molecular role players contributing to risk, thus advancing our understanding of potential therapeutic targets. This model may also be employed going forward, for drug discovery approaches. In terms of application, it is important to note that reported comorbidities develop over time in the affected human offspring, with a complex aetiology also affected by other contributing factors. Thus, a simple validation exercise in zebrafish using knock-in or knock-out models of differentially regulated proteins observed in the current study, is unlikely to result in overt phenotypic changes. Rather, potential modulation of these proteins or related genes should be assessed for their value as potential biomarkers of risk in relevant human populations. For example, clinical studies involving offspring exposed to SER/DES during pregnancy are crucial for corroborating our findings, particularly by assessing the expression of these downregulated genes or their protein products in blood samples. Existing studies primarily report phenotypic outcomes, such as altered behaviours and organ malformations, without elucidating the underlying mechanisms responsible for these observations. This gap limits the identification of therapeutic targets that could potentially mitigate these risks. Therefore, a deeper understanding of the molecular pathways involved is needed to inform more effective intervention strategies. This may also include evaluating cardiac performance in offspring exposed to SER and DES *in utero*.

### Limitations

In terms of methodology, we interpreted relative hypolocomotion and decreased SERT expression levels as indicative of a therapeutic SSRI effect. Addition of a positive control pharmaceutical may have strengthened this interpretation. Similarly, confirmation of current data via proteomics analysis in similar mammalian models, and validation of proteomics outcomes through assessments at protein level to provide information on system-specific dysregulation, could add further value in this context.

A potential limitation to the translation of current data is the fact that sex was not a consideration, due to the absence of sex-specific phenotypes in larval zebrafish. It would therefore be of interest to validate the longevity of the observed modulations in juvenile zebrafish, where sex-specificity may also be elucidated.

Finally, although the relatively young age of larvae used was an appropriate choice for simulation of a developing foetus, assessments at a somewhat more developed age may facilitate more in-depth analyses of the serotonergic system. In the current study evaluation of SERT expression was also attempted in the gut given the relatively higher abundance of serotonin in the gut. However, given the still underdeveloped gut, expression levels were relatively low and thus not reported here. To facilitate comparisons between gut and brain dysregulation, we recommend that slightly older larvae be used.

## Conclusion

SER and DES at umbilical cord blood equivalent concentrations induced dysfunction in the developing zebrafish serotonergic system and altered behaviours in standardised assays, underscoring the high risk of relative 5-HT toxicity in exposed offspring. Proteomic data suggest that DES, rather than the parent drug, SER, is primarily responsible for the adverse clinical outcomes reported following gestational SSRI use. This metabolite generally exhibits higher plasma exposure than the parent drug, including in umbilical cord blood, which indicates an increased risk to the developing foetus in mothers taking SER.

Finally, the close alignment of current data to that reported from human cohorts, suggests the zebrafish as potential model organism for research in this niche.

## Supplementary Information

Below is the link to the electronic supplementary material.

Supplementary Material 1

## Data Availability

All data is presented within the manuscript or supplementary information file. Raw data may be requested from the corresponding author.

## Abbreviations

5-HT: Serotonin
ASD: Autism spectrum disorders
CLUH: Clustered mitochondrial protein homolog
DDX39A: ATP-dependent RNA helicase
DES: Desmethylsertraline
DHRS11: Dehydrogenase/reductase SDR family member 11
DMSO: Dimethylsulfoxide
DRAP1: Drf1-associated co-repressor
GO: Gene ontology
HED: Human equivalent dose
Hpf: Hours post fertilisation
HSPB1: Heat shock protein beta 1
LDTT: Light-dark transition test
MATN1: Cartilage matrix protein
MYL2: Myosin regulatory light chain alpha 2
PHB1: Prohibitin 1
PPIs: Protein-protein interactions
PTU: 1-phenyl 2-thiourea
ROI: Region of interest
RPL30: Ribosomal protein L30
SER: Sertraline
SERT: Serotonin transporter
SSRIs: Selective serotonin reuptake inhibitors
