# Supplementary material for: The sertraline metabolite, desmethylsertraline, may be implicated in adverse outcomes reported after gestational sertraline use: insights from a study in zebrafish
Source: Pharmacol Rep. 2025 Jul 28;77(5):1374–88. doi: 10.1007/s43440-025-00765-y (PMC12443929; doi:10.1007/s43440-025-00765-y)
Supplement: Supplementary file 1 — Supplementary Material 1 [file 43440_2025_765_MOESM1_ESM.docx]

**The sertraline metabolite, desmethylsertraline, may cause adverse outcomes reported after gestational sertraline use: insights from a study in larval zebrafish**

Cassius M. Phogole^1^, Lesha Pretorius^2^_,_ Tracy Kellermann^1^_,_ Maré Vlok^3^ _,_ Carine Smith^2*^

**Supplementary materials**

**Materials**

Both SER (Sertraline hydrochloride (98% purity); cat no: 4-DAR-9-1) and DES (*rac*-cis-N-desmethylsertraline hydrochloride (97% purity); cat no: 1-MOZ-80-1) were purchased from Toronto Research Chemicals (TRC), Canada. Phosphate-buffered saline (PBS), tris 2-carboxy ethyl phosphine (TCEP), tris (hydroxymethyl) aminomethane hydrochloride (Tris-HCl), sodium chloride (NaCl), sodium dodecyl sulfate (SDS), ammonium bicarbonate, lysozyme, trifluoroacetic acid (TFA), iodoacetamide (IAA), dithiothreitol (DTT), 1-phenyl 2-thiourea (PTU), and dimethylsulfoxide (DMSO) were procured from Sigma-Aldrich (Germany). Ammonium acetate and trifluoroacetic acid (TFA) were obtained from Fisher Chemical (Spain). Trypsin and a 12-tube magnetic separation rack were purchased from New England Biolabs (United States). Acetonitrile (ACN) was obtained from Romil Pure Chemistry (South Africa). MagReSyn® HILIC magnetic microparticles was procured from Separations (South Africa). Indexed Retention Time (iRT) standards Kit was purchased from Biognosys (United States).

**Treatment exposure**

Zebrafish embryos of less than 4 hpf were randomly assigned to a control group and two treatment groups of SER and DES, respectively. Treatment concentrations were determined by translating the median values of actual human umbilical cord blood concentrations of SER and DES reported in mothers at delivery treated with SER during pregnancy [29]. The conversion formula applied is published elsewhere [28]. Initial stock solutions of SER and DES were prepared at 1 mg/mL in DMSO before serial dilution with E3 medium, resulting in negligible final DMSO levels. The final concentrations of SER and DES to which the embryos were exposed were 25 ng/mL and 20 ng/mL, respectively. The control group was maintained in E3 medium free of SER and DES. Treatment exposure lasted for 114 hours and media were refreshed daily. Maintenance conditions included a 14-hour light/10-hour dark cycle, with temperature maintained at 28°C for the entire experiment duration. At experiment endpoint (118 hpf), subsets of larvae (n = 55 per group, in triplicate) were pipetted into 1x PBS and immediately euthanized by snap-freezing in liquid nitrogen. The samples were then stored at -80°C until further processing.

**Behavioural assay**

At 118 hpf, larvae (n = 20-24 per group, repeated in three separate experiments) were transferred into 96-well plates and subjected to the light-dark transition test (LDTT), following a standard protocol described elsewhere [28]. Briefly, the test started with acclimatization in perceived darkness (infrared light) for 10 minutes (min) each, followed by a light-dark transition cycle (bright white light exposure and subsequent perceived darkness for 10 min each). The light-dark transition elicits a robust hyperlocomotion response during the last period of darkness. The total activity levels (distance moved over time) were quantified by automated activity tracking (DanioVision video-tracking system equipped with EthoVision software XT 15 (Noldus, Wageningen, Netherlands)).

**Whole-mount immunofluorescent staining**

Whole-mount immunofluorescent staining was performed using an established protocol [90] to assess expression levels of SERT. Briefly, at the experimental endpoint (118 hpf), larvae were euthanised using tricaine overdose, before overnight fixation with 4% paraformaldehyde (PFA) at 4˚C. Larvae were then washed with phosphate-buffered saline (PBS) twice prior to permeabilization with pre-chilled acetone for 7 min at -20˚C. Afterward, larvae were washed twice with PBS before being incubated at room temperature for 3 hours with a blocking buffer comprised of 20% foetal bovine serum (FBS), 5% donkey serum, and 0.2% Triton-X in PBS. The larvae were then incubated overnight at 4˚C with primary antibody diluted with 20% blocking buffer in PBS. The goat anti-SERT primary antibody (Abcam; cat no: ab13030) was prepared as 1:10 dilution. Subsequently, the larvae were washed with 0.1% Tween 20 in PBS (PBS-T) three times for 5 min each, before a final overnight incubation with secondary antibodies at 4˚C. The secondary antibody was a 1:100 Alexa Fluor®488 donkey anti-goat (Abcam; cat no: ab150129) prepared with 20% diluted blocking buffer. Finally, the larvae were washed three times, 5 min each, with PBS-T before a final washing step with a storing buffer (PBS). Image acquisition was done using brightfield and fluorescence microscopy on a Nikon® ECLIPSE Ti2 inverted microscope fitted with a 10x objective. Images were captured using NIS-Elements software (version D v 5.30.02). The ImageJ software (version 2.1.0) was used to quantify immunofluorescence intensity in the region of interest (forebrain). One image was captured per individual larva, and at least six larvae were included in the statistical analysis following an outlier test. The mean of multiple images per treatment group was used for statistical analysis.

**Proteomics**

*Sample preparation and protein digestion*

Fifty-five pooled larvae per sample were initially stored in 1 mL of 1x PBS. The storage buffer was subsequently diluted with a 10x stock solution containing Tris-HCl, SDS, and NaCl. The final concentrations achieved were 100 mM Tris-HCl (pH 8.0), 1% SDS, and 100 mM NaCl. The samples were then mixed for 5 min with end-over-end mixing before sonication for three minutes and vortexing at high speed for 30 s. This process was repeated 3 times before the samples were centrifuged at 12 000 x g for 10 min. The sonication and vortex steps were repeated before being centrifuged again. After centrifugation the samples were adjusted to 100 mM ammonium acetate with 1 M ammonium acetate pH 4.5. The sample was again mixed end-over-end for 5 min before being adjusted to 15% ACN (v/v).

The samples were again mixed before the addition of 20 µL of Magresyn beads (unequilibrated). The samples and the magnetic beads were mixed end-over-end overnight to allow protein binding. The next morning the samples were adjusted to 30% ACN and mixed for a further three hours. After the final binding step was completed, the beads were allowed to settle on-bench for 30 min before being placed on magnets for final separation from the extraction solution. The extraction solution was removed, and an on-bead reduction was performed by adding 50 µL 100 mM ammonium acetate, pH 4.5, containing 15% ACN and 5 mM TCEP. The samples were incubated at room temperature for 1 hour before the addition of 20 mM IAA. This mixture was incubated in the dark for 30 min. The carboxymethylation reaction was quenched by adjusting the reaction mixture to 20 mM DTT.

The reaction mixture was removed, and the beads washed twice with 200 µL ACN:water (95:5, v/v). After washing, the beads were re-suspended in 50 µL 50 mM ammonium bicarbonate containing trypsin in a theoretical 1:50 ratio (1 µg trypsin per sample). The samples were incubated overnight at 37°C. The solution containing the protein digest was removed and kept and the beads washed with 50 µL of 1% TFA in water. The wash was combined with the digest solution and centrifuged for 10 min at 12 000 x g. The supernatant was removed and placed in HPLC glass inserts (240 µL inserts) and dried under vacuum. The dried peptides were dissolved in 30 µL MilliQ water for concentration determination using a nano-drop spectrophotometer 7415 (Jenway) at absorbance wavelength of 214 nm.

*Liquid chromatography (Dionex nano-RSLC) and mass spectrometry*

Chromatographic separations were performed on a Thermo Scientific Ultimate 3000 RSLC instrument using the following columns: Thermo Scientific C18 trap column (20 mm x 100 µm) and a Waters CSH C18 column (1.7 µm, 25 cm x 75 µm), respectively. The loading solvent consisted of 2% ACN in MilliQ water containing 0.1% FA where solvents A and B were water with 0.1% formic acid and 100% ACN, respectively. Samples from the autosampler set at 7ºC were transferred onto the trap column. The flow rate of the loading solvent was 2 mL/min, for 3 min before being eluted onto the analytical column at 300 nL/min, and the column thermostat was maintained at 45ºC. For elution from the column, a linear gradient was employed with the following composition: 5% to 30% B over 60 minutes, followed by 30% to 50% B from 60 to 80 minutes.

Data independent acquisition (DIA) mass spectrometry analysis was performed using a Thermo Scientific Fusion mass spectrometer, equipped with a Nanospray Flex ionisation source. The prepared samples were introduced through a stainless-steel nano-bore emitter. Data were collected in a positive mode with spray voltage set 2.0 kV and ion transfer capillary at 290°C. Polysiloxane ions, at 445.12003 m/z, were used for internal calibration of the spectra. For MS1 scans, the Orbitrap detector was set to a resolution of 60 over a scan range of 375 – 1500 m/z, with an automatic gain control (AGC) target at 4 E5 and a maximum injection time of 50 ms. Data acquisition was conducted in profile mode. Monoisotopic precursor selection was used for ion charges +2 to +7 with an error tolerance ± 10 ppm for MS2 acquisitions. Precursor ions were excluded from fragmentation for 60 s. In higher-energy C-trap dissociation (HCD) mode, precursor ions were selected for fragmentation using the quadrupole mass analyser with HCD energy set to 30%. Three windows were used to scan precursor ions: 355 – 555 m/z, 555 – 755 m/z, and 755 – 955 m/z, with an isolation window of 10 m/z, and an overlap of 1 m/z. Fragment ions were detected using the Orbitrap mass analyser set to a resolution 30, 000. The AGC target was set to 5 E4, and the maximum injection time to 100 ms. Data acquisition was performed in centroid mode.

*Data analysis: Database interrogation*

The raw files generated by the mass spectrometer were combined by treatment replicates (n = 3) and subsequently imported into the FragPipe computational platform (version 22.0). Data were processed using the DIA_SpecLib_Quant workflow, with strict trypsin cleavage allowed. Two missed trypsin cleavages were permitted. Deamidation (NQ), oxidation (M), and acetylation of protein N-terminal were allowed as dynamic and thiomethyl as static modifications, respectively. The mass tolerance for precursors was set to ±10 ppm, while the fragment mass tolerance was set to 0.02 Da. Peptide length and peptide mass range were set at ranges of 7 – 50 and 500 – 5000, respectively. Peptide validation was conducted using the Target-Decoy PSM validator node, and DIA quantification was performed using DIA-NN.

Database interrogation was initially performed using the individual UniProt databases (www.uniprot.org) for *Danio rerio* (zebrafish) and *Homo sapiens* (humans), as zebrafish exhibit conserved vertebrate biology and share about 70% genetic similarity with humans [37,38]. Statistical analyses were conducted using FragPipe-analyst as follows: the limma package from R Bioconductor was used to generate a list of differentially expressed proteins for each pairwise comparison. A cutoff of an adjusted p-value of 0.01 (using the Benjamini-Hochberg method) along with a log2-fold change of 1 was applied to determine significantly regulated proteins in each pairwise comparison. Additionally, protein-protein interactions were assessed using the STRING App in Cytoscape (version 3.9.1). Finally, the functionality of the protein was analyzed at the molecular, biological, and pathway levels using Gene Ontology (<http://www.bioinformatics.com.cn/>).

**Supplementary results**

**Survival and hatching rate**

While hatching seemed to be delayed for most SER and DES groups at 48 hpf, no differences persisted after 72 hpf (Figure S1). Similarly, total mortality indicates higher levels of mortality for all exposure groups, however, this was only observed after 24 hpf, whereafter no more deaths were recorded in any groups.

**
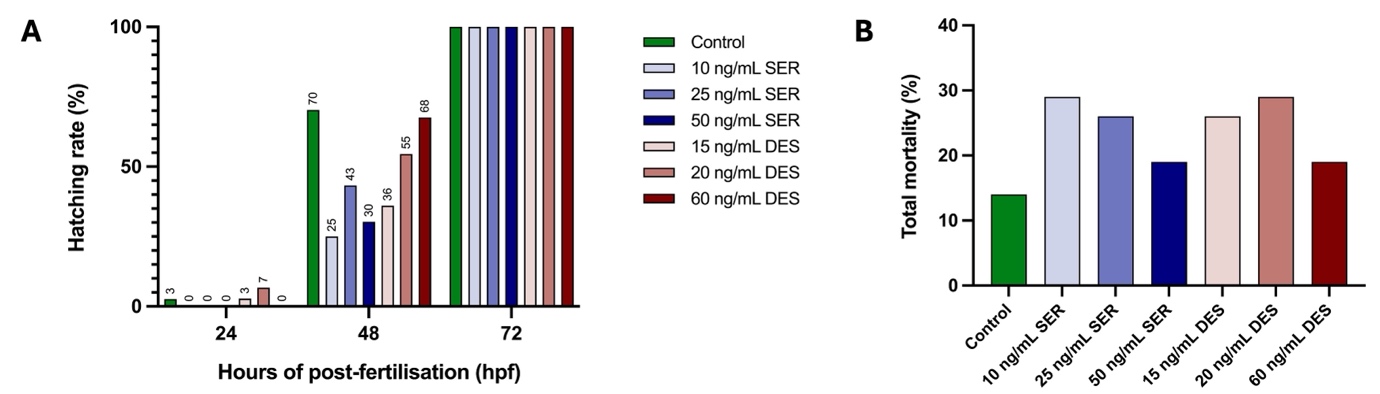
**

**Figure S1.** The effect of sertraline (SER) and desmethylsertraline (DES) exposure (at concentrations equivalent to cord blood levels) on hatching rate (A) and total mortality (B) in zebrafish embryos.

**Immunohistochemistry (SERT protein expression)**

We provide additional representative images for the IHC images used for SERT protein expression levels.


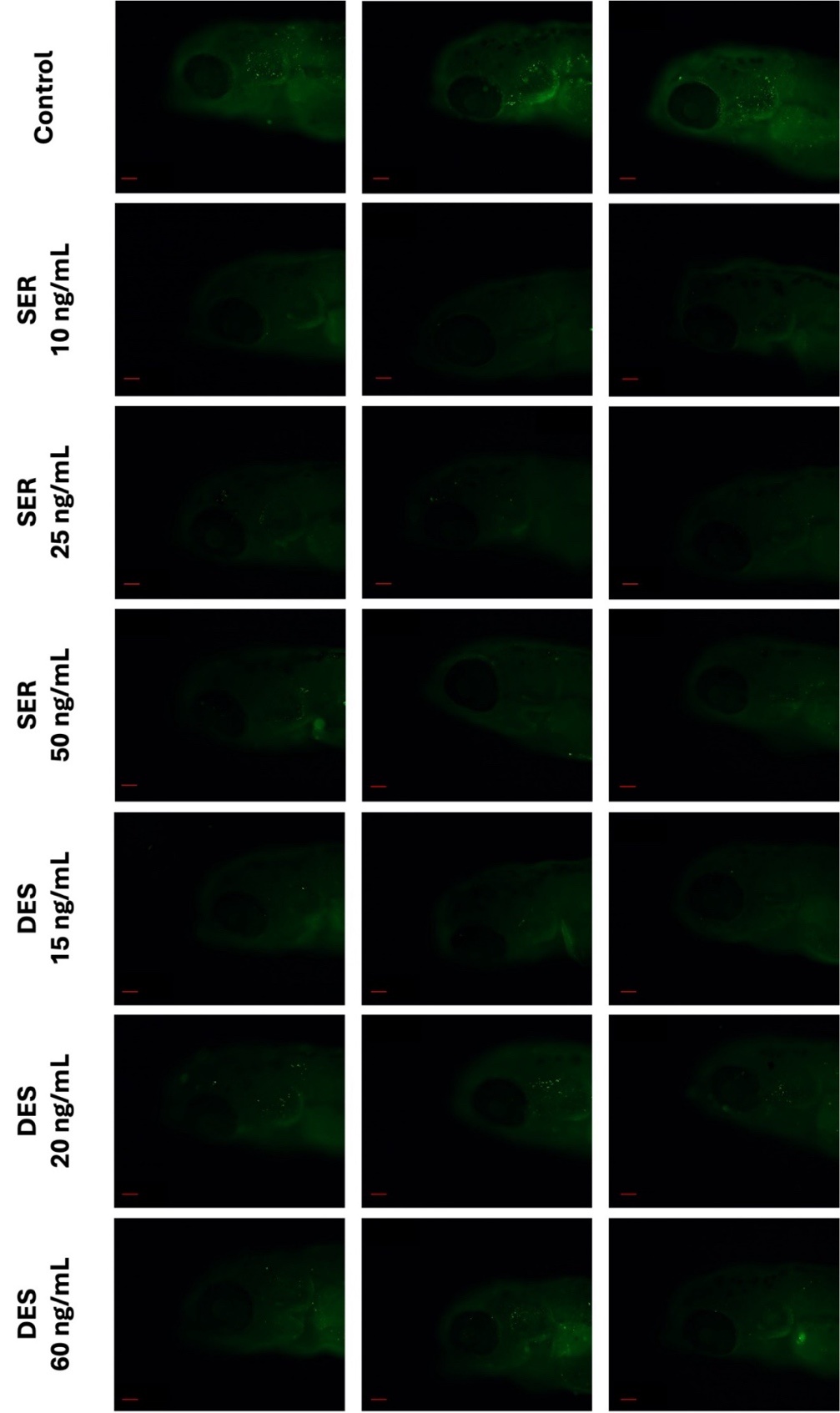


**Figure S2.** Additional representative images of serotonin transporter (SERT) expression levels following prolonged exposure (4 to 120 hpf) to sertraline (SER) and desmethylsertraline (DES) at concentrations equivalent to cord blood levels, to complement data presented in manuscript Figure 2. Larvae were imaged using a 10x objective lens at 100x magnification; scale bar = 50 μm.

**Proteomic analysis - additional detail**

Principal component analysis (PCA), a dimensionality reduction method that transforms a large set of variables into a smaller set while preserving most of the original information, revealed good separation among the studied samples. The first principal component (PC1) represents the direction that accounts for the maximum variance in the data, while the second principal component (PC2) accounts for the second highest variance. Together, PC1 and PC2 accounted for 44.6% and 29.2% of the total variability, respectively, in the triplicate samples (Figure S3). The analysis clearly shows that the control group and the DES-treated group are well-separated along both PC1 and PC2, indicating that DES had a significant impact on the biological samples. The reproducibility within both groups was high, with only a minor divergence observed in one replicate from the DES-treated group.


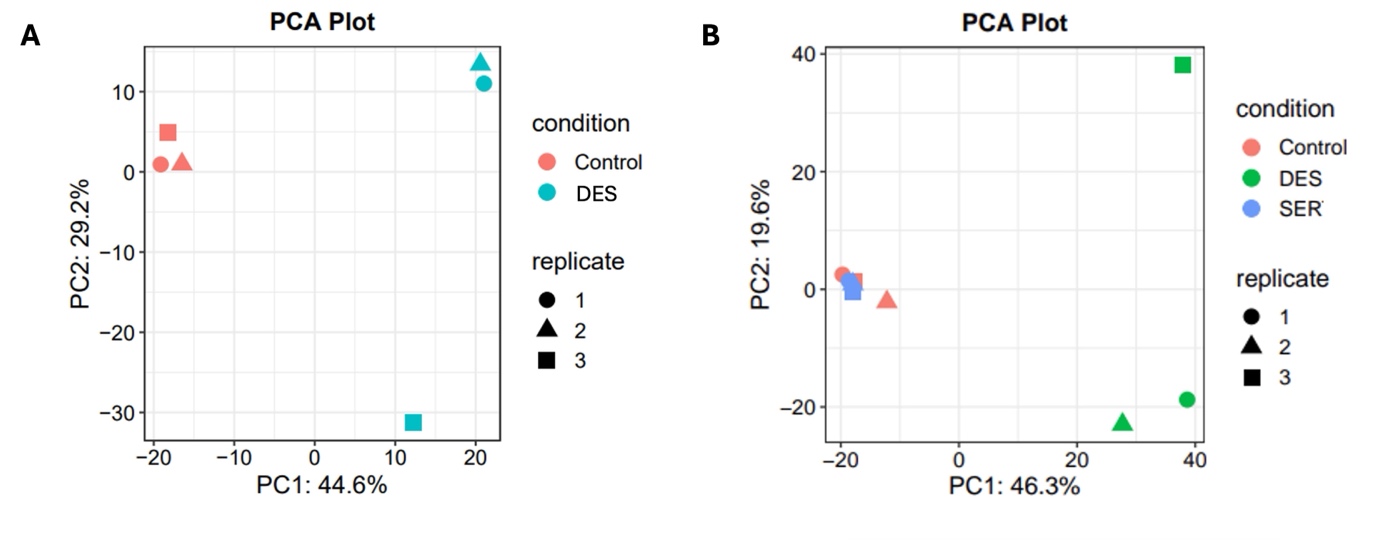


**Figure S3.** Principal component analysis (PCA) patterns of zebrafish larval proteomes, interrogated using human (A) and zebrafish (B) databases, from triplicate samples of control and treatment groups (114hr exposure to sertraline (SER; 25 ng/mL) or desmethylsertraline (DES; 20 ng/mL)), used to assess inter-sample and intra-sample variability and reproducibility.

A heatmap plot (Figure S4) represents an overview of the expression of all significant (differentially expressed) proteins (rows) in all samples (columns). The red and blue colours in the condition represent control and DES samples, respectively. This analysis was conducted to identify patterns and correlations among the samples. As illustrated, the control groups exhibit more similar features (proteins) compared to the treatment group, where the protein levels are significantly lower.


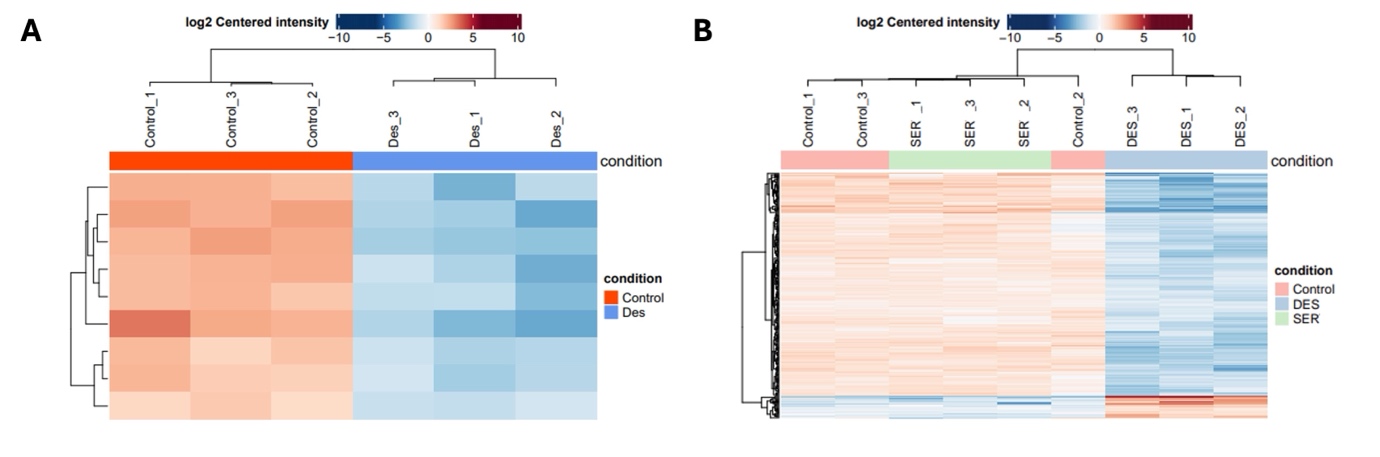


**Figure S4.** Proteome profiles of zebrafish larvae (n = 55 per pooled sample, n = 3 per treatment group) exposed to sertraline (SER; 25 ng/mL) and desmethylsertraline (DES; 20 ng/mL) for 114 hours, compared to the control group (interrogated using human (A) and zebrafish (B) databases). The color scale at the top represents log2-centered intensity values, ranging from blue (indicating lower intensity or protein expression) to red (indicating higher intensity or protein expression). The heatmap is based on hierarchical clustering (p<0.01). The dendrograms at the top and left of the heatmap represent the hierarchical clustering of samples and features, respectively.

**Higher resolution images of results from Markov Cluster Algorithm (MCL) clustering and GO (Gene Ontology) enrichment analysis of differentially regulated proteins (supplement to Figures 4,5 and 7)**

*
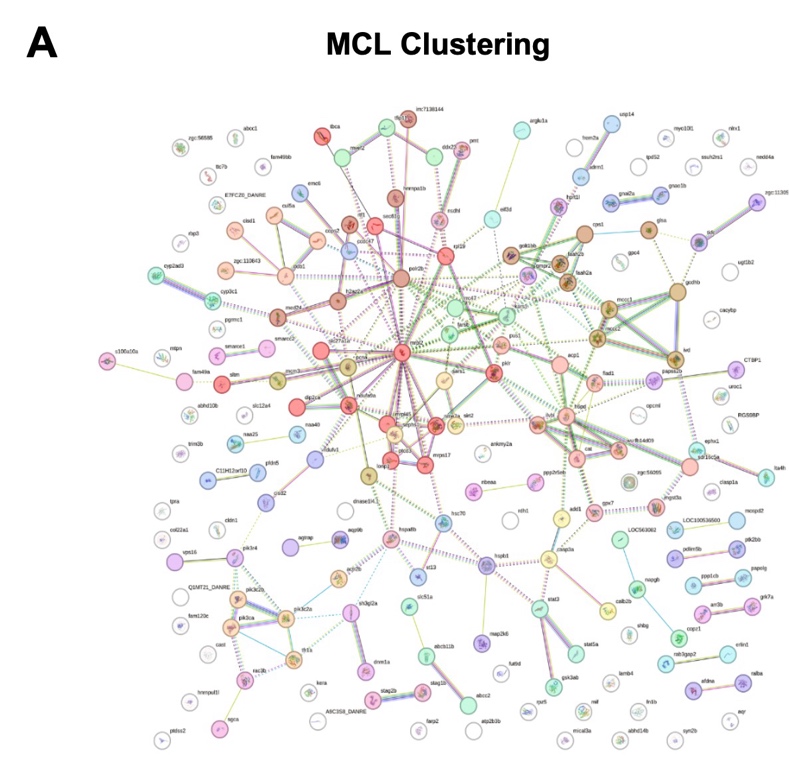
*

*
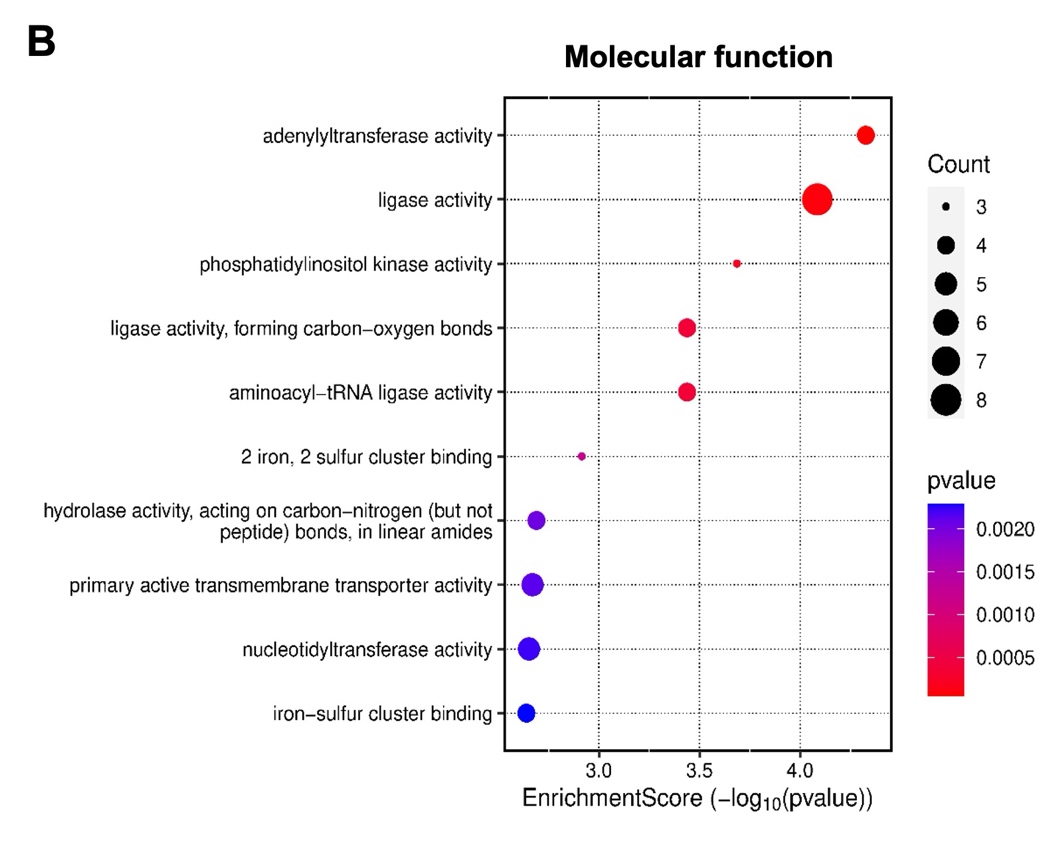
*

*
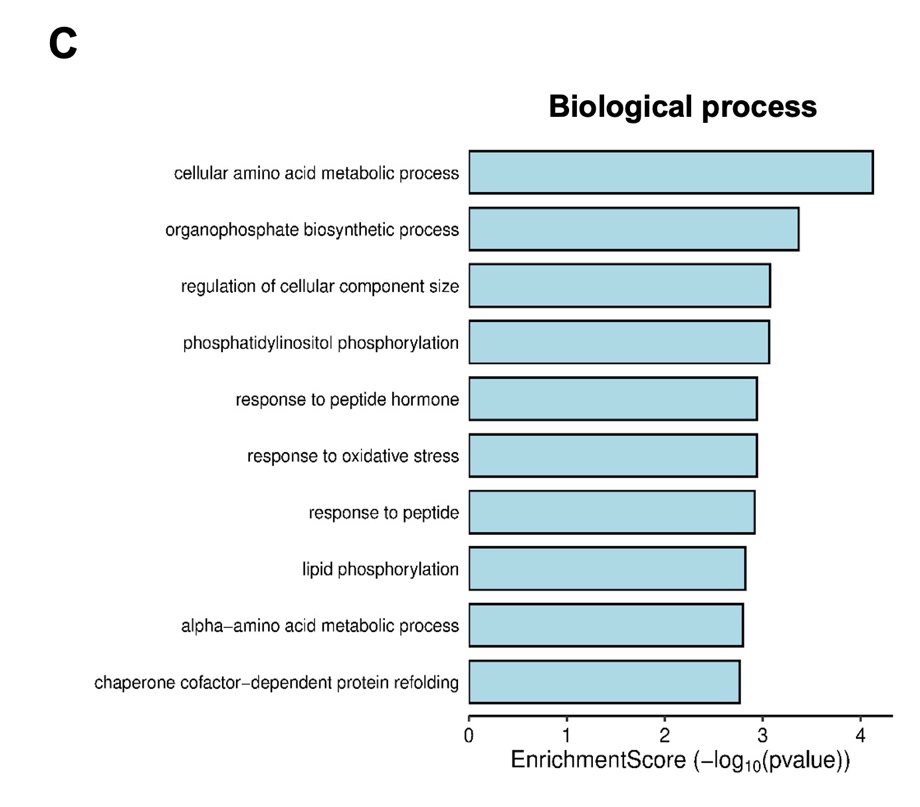
*

*
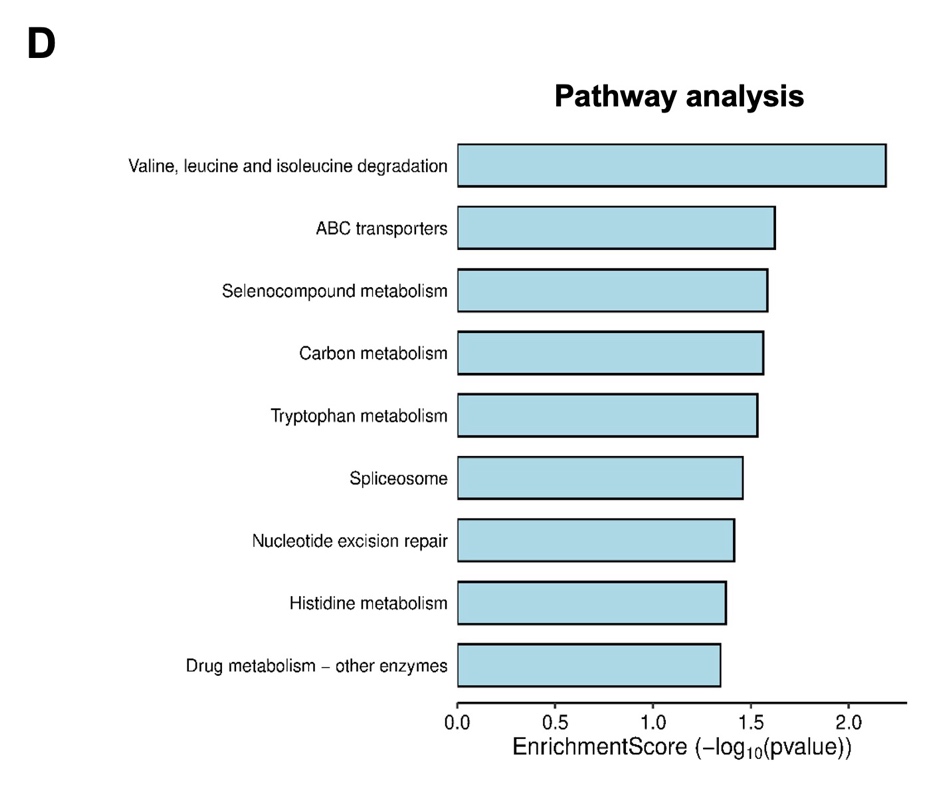
*

**Figure S5.** Markov Cluster Algorithm (MCL) clustering results from the STRING database and GO (Gene Ontology) enrichment analysis of upregulated proteins (interrogated using a zebrafish database) in zebrafish larvae treated with desmethylsertraline (DES; 20 ng/mL) for 114 hours (refer to Table S1 for specific proteins). Protein-protein interaction (PPI) regulatory network (A): Nodes represent proteins, and edges denote protein-protein associations. Green, pink, and black lines indicate text-mining, experimentally determined interactions, and co-expression, respectively. Refer to supplementary material for identification of all identified differentially regulated proteins. Molecular functions (B), biological processes (C), and pathway analysis (D) were identified through GO enrichment analysis. The x-axis represents the -log10 of p-values or enrichment scores, while the y-axis displays up to 10 identified significant items, ranked in increasing order of significance.

**
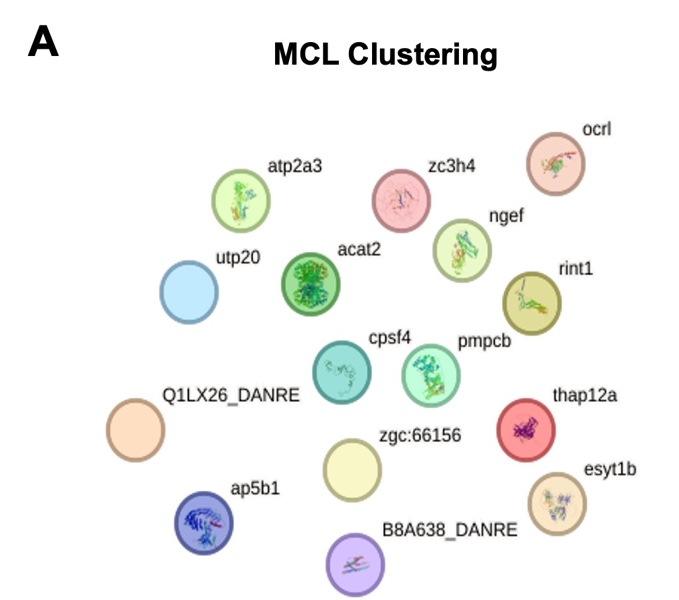
**

**
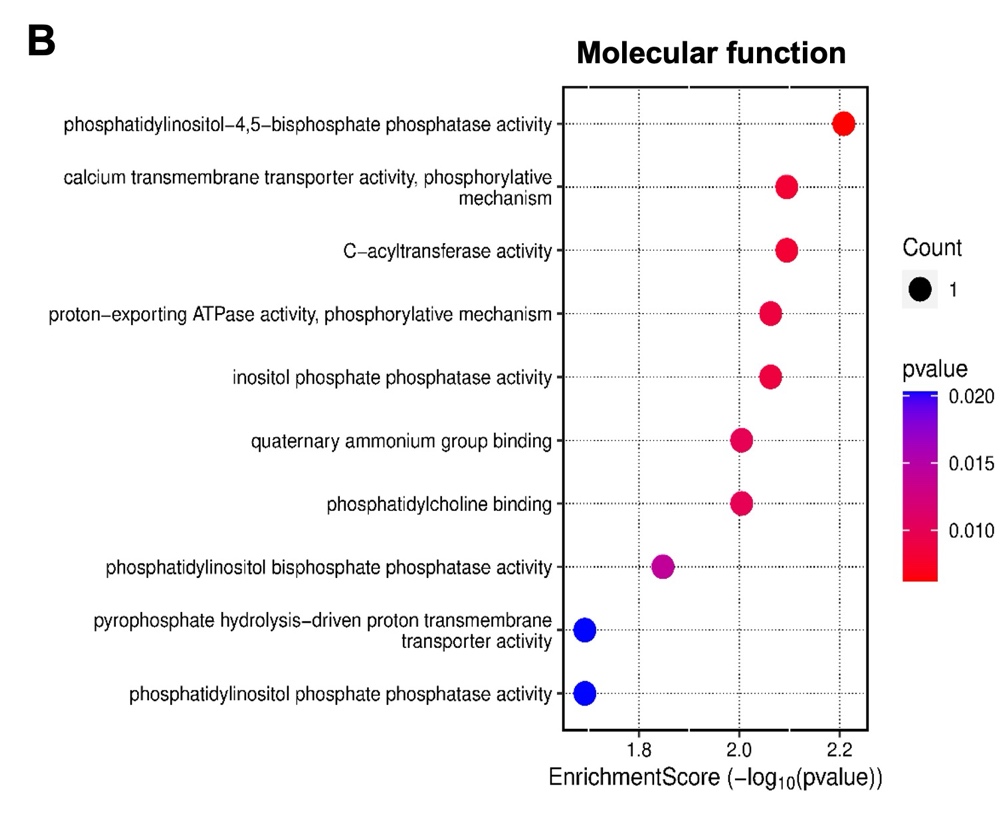
**

**
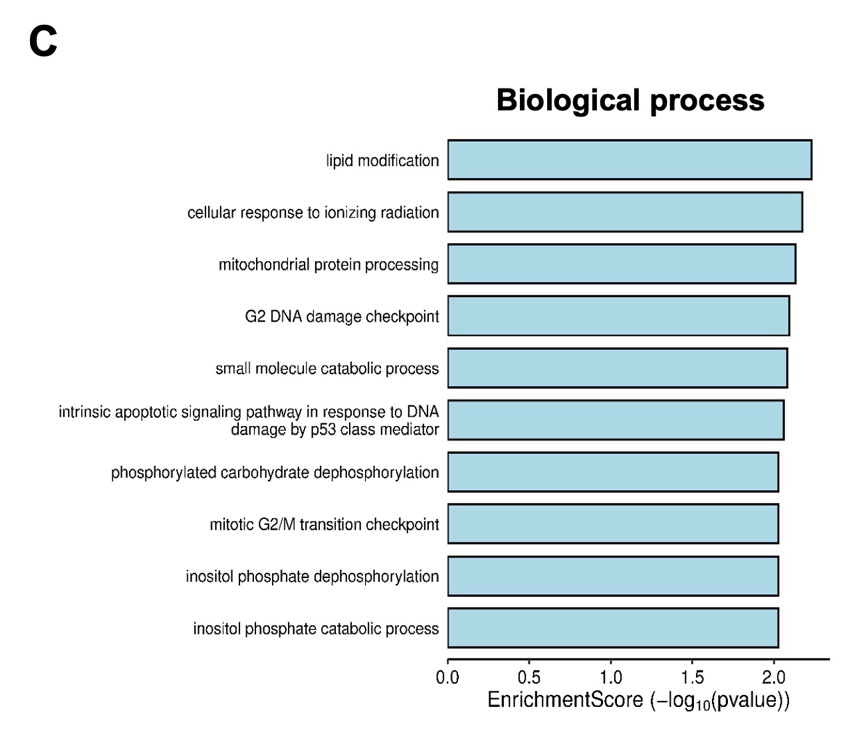
**

**
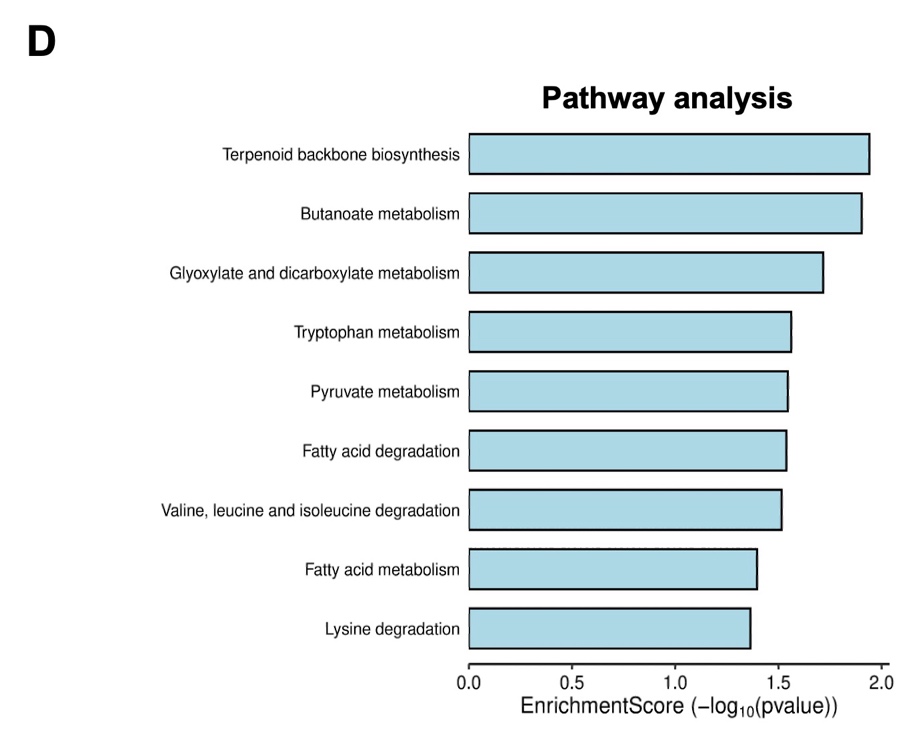
**

**Figure S6.** Markov Cluster Algorithm (MCL) clustering results from the STRING database and GO (Gene Ontology) enrichment analysis of downregulated proteins (interrogated using a zebrafish database) in zebrafish larvae treated with desmethylsertraline (DES; 20 ng/mL) for 114 hours (refer to Table S2 for specific proteins). Protein-protein interaction (PPI) regulatory network (A): Nodes represent proteins, and edges denote protein-protein associations. Green, pink, and black lines indicate text-mining, experimentally determined interactions, and co-expression, respectively. Refer to supplementary material for identification of all identified differentially regulated proteins. Molecular functions (B), biological processes (C), and pathway analysis (D) were identified through GO enrichment analysis. The x-axis represents the log10 of p-values or enrichment scores, while the y-axis displays up to 10 identified significant items, ranked in increasing order of significance.

**
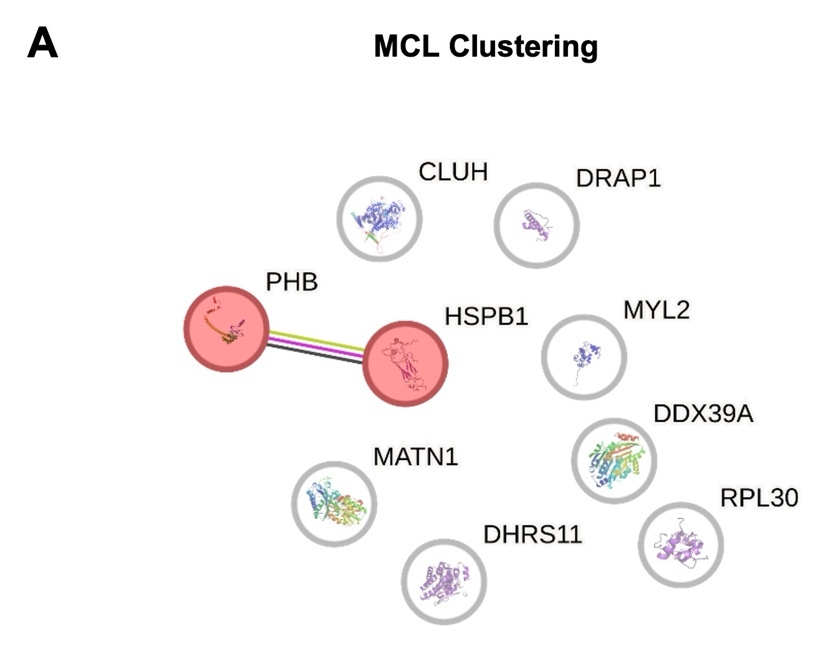
**

**
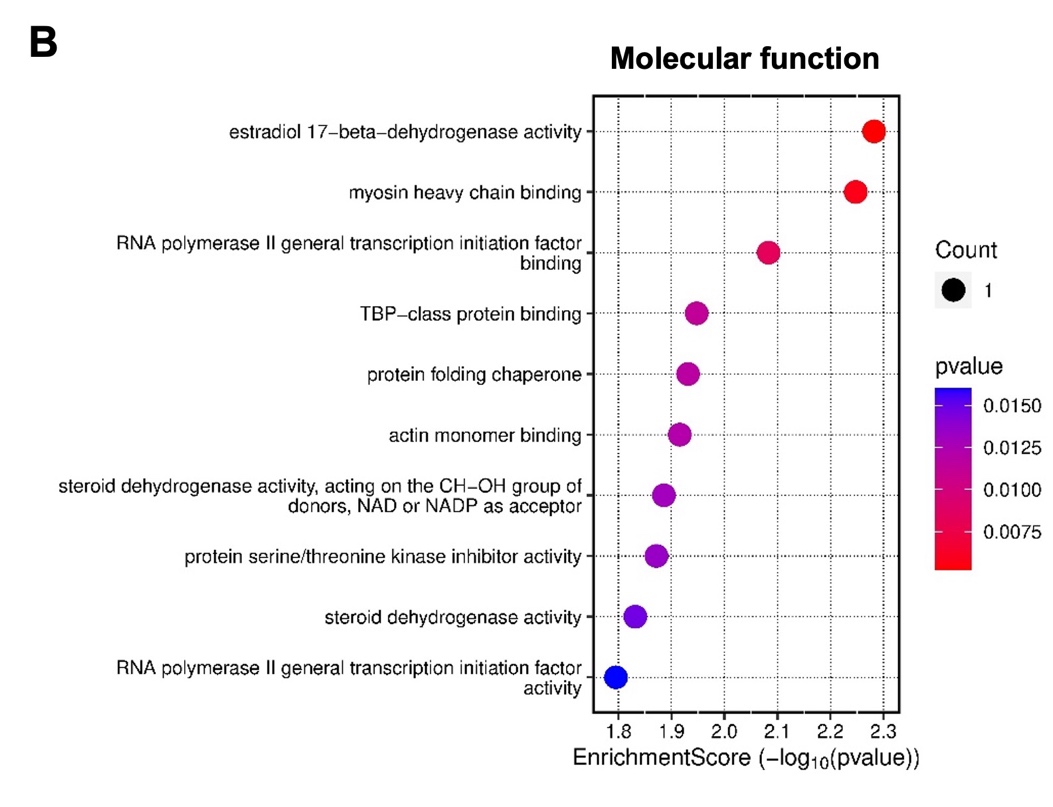
**

**
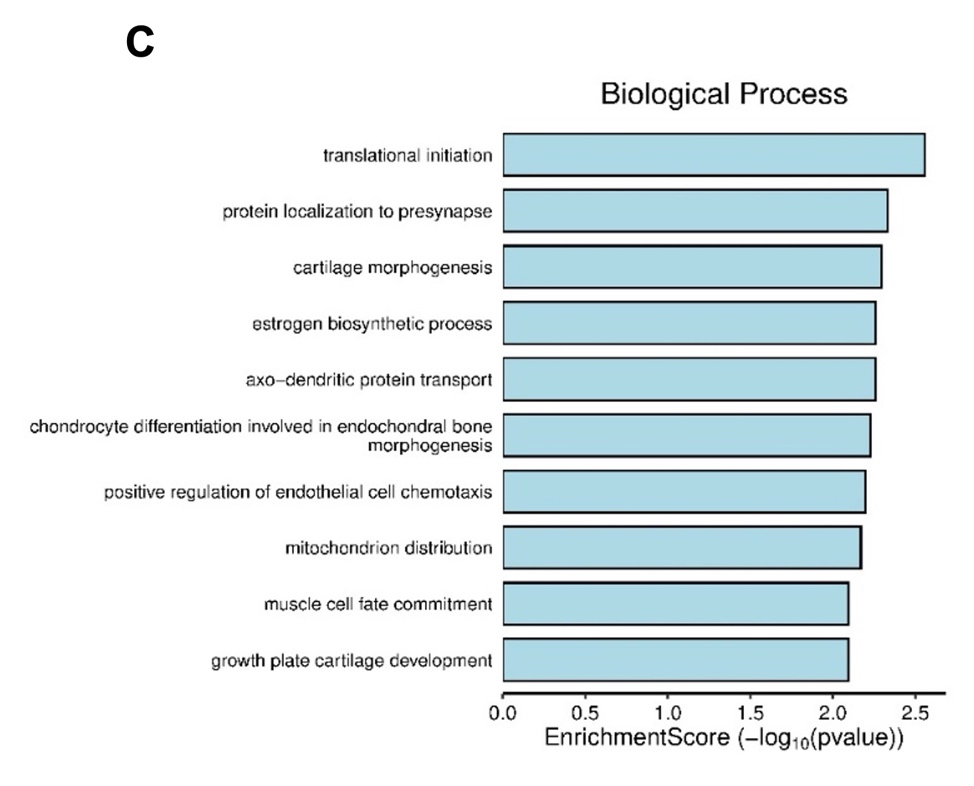
**

**
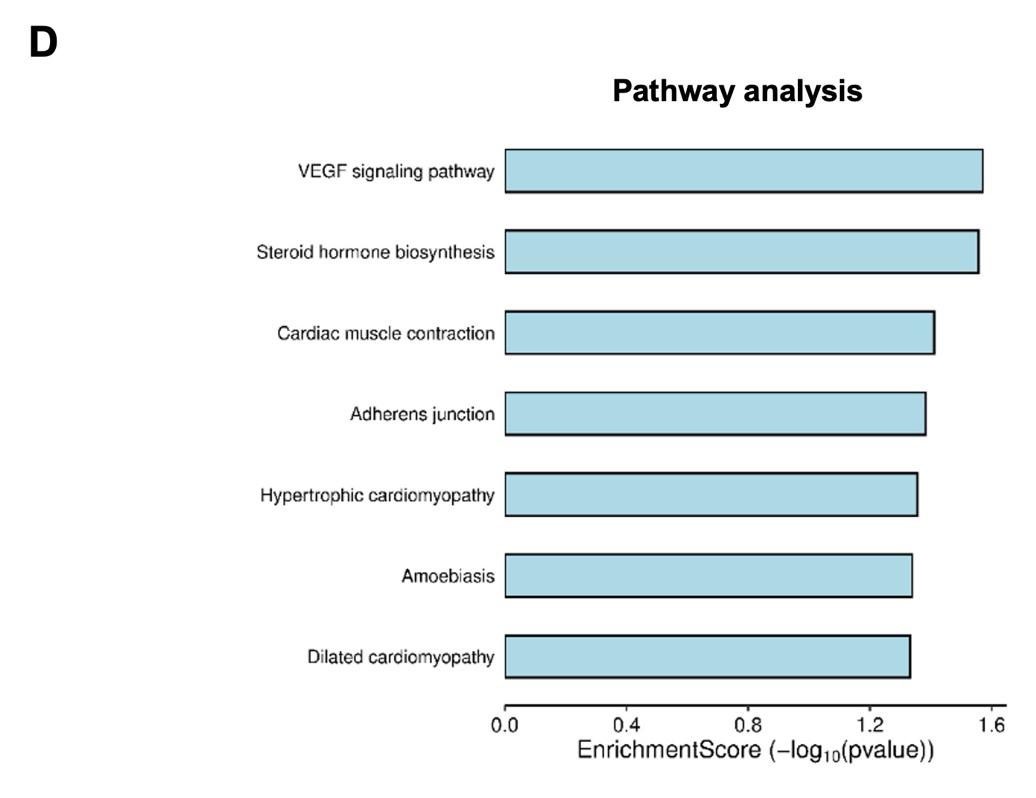
**

**Figure S7.** Markov Cluster Algorithm (MCL) clustering results from the STRING database and GO (Gene Ontology) enrichment analysis of downregulated proteins (interrogated using a human database) in zebrafish larvae treated with desmethylsertraline (DES; 20 ng/mL) for 114 hours (refer to Table S3 for specific proteins). Protein-protein interaction (PPI) regulatory network (A): Nodes represent proteins, and edges denote protein-protein associations. Green, pink, and black lines indicate text-mining, experimentally determined interactions, and co-expression, respectively. Molecular functions (B), biological processes (C), and pathway analysis (D) were identified through GO enrichment analysis. The x-axis represents the log10 of p-values or enrichment scores, while the y-axis displays up to 10 identified significant items, ranked in increasing order of significance.

**List of differentially regulated proteins (DRPs) identified (supplement to Figures 4,5 and 7)**

**Table S1.** DRPs identified using zebrafish protein database (upregulated proteins).

| **Gene Names** | **Protein names** | **Log2-fold change**  **(upregulated)** |
| --- | --- | --- |
| cyp46a1.5 zgc:123299 | Uncharacterized protein LOC641477 isoform 1 (Zgc:123299) | 2.98 |
| mccc1 si:dkey-37m8.6 zgc:154106 | Methylcrotonoyl-CoA carboxylase subunit alpha, mitochondrial (EC 6.4.1.4) (Methylcrotonyl-CoA carboxylase subunit) | 4.29 |
| lonp1 LONP1 | Lon protease homolog, mitochondrial (EC 3.4.21.53) | 2.87 |
| syn2b zgc:123199 | Synapsin IIb (Synapsin-2b) | 2.18 |
| fam49a fam49ab sb:cb866 si:ch211-223o11.1 si:ch211-89p3.1 zgc:55309 znf395 | Family with sequence similarity 49 member A (Uncharacterized protein LOC378872 isoform 3) | 2.08 |
| stag2b | Cohesin subunit SA (SCC3 homolog) (Stromal antigen) | 2.99 |
| myg1 fa14e09 im:6894200 wu:fa14e09 zgc:136866 | Myg1 exonuclease (UPF0160 protein MYG1, mitochondrial) | 3.07 |
| rdh1 RDHB | Retinol dehydrogenase 1 | 2.5 |
| mospd2 wu:fc33h03 wu:fc48a06 wu:fe36a12 zgc:92558 | Motile sperm domain-containing 2 (Motile sperm domain-containing protein 2) | 1.62 |
| pik3c2a | Phosphatidylinositol 4-phosphate 3-kinase C2 domain-containing subunit alpha isoform X1 (Phosphatidylinositol-4-phosphate 3-kinase, catalytic subunit type 2 alpha) | 2.26 |
| dnase1l4.1 cb217 dnase1l3 zgc:101000 | Deoxyribonuclease | 1.89 |
| ppp1cb ik:tdsubc_2f2 si:zc214p16.4 wu:fa09h01 wu:fa11e06 xx:tdsubc_2f2 | Serine/threonine-protein phosphatase PP1-beta catalytic subunit (EC 3.1.3.16) | 3.58 |
| polr2b zgc:109952 | DNA-directed RNA polymerase subunit beta (EC 2.7.7.6) | 2.16 |
| myef2 zgc:123351 zgc:152722 | Myelin expression factor 2 | 3.16 |
| acp1 im:6910498 zgc:110844 | Low molecular weight phosphotyrosine protein phosphatase (LMW-PTP) (LMW-PTPase) (EC 3.1.3.2) (EC 3.1.3.48) (Low molecular weight cytosolic acid phosphatase) | 3.79 |
| mrpl2 im:6908224 | 39S ribosomal protein L2, mitochondrial precursor (Mitochondrial ribosomal protein L2) | 2.51 |
| hnrnpa1b hnrnpa1 hnrpa1 | Heterogeneous nuclear ribonucleoprotein A1b | 3.78 |
| pik3r4 | non-specific serine/threonine protein kinase (EC 2.7.11.1) | 1.78 |
| hnrnpul1l | Heterogeneous nuclear ribonucleoprotein U-like protein 1 | 2.27 |
| abcb11b | Bile salt export pump | 3.2 |
| trim3b zgc:153967 | RING-type E3 ubiquitin transferase (EC 2.3.2.27) | 5.03 |
| LOC101883107 | Integrase catalytic domain-containing protein | 4.48 |
| tpd52 si:ch211-203l9.2 si:ch211-203l9.3 | Tumor protein D52 isoform X1 | 2.17 |
| cast sb:cb966 zgc:194249 | Calpastatin (Calpain inhibitor) | 1.92 |
| stat3 STAT3b1 STAT3b2 wu:fc15d02 wu:fl59g06 z-Stat3 | Signal transducer and activator of transcription | 3.1 |
| smarce1 baf57 fb33d02 fb54d11 wu:fb33d02 wu:fb54d11 | SWI/SNF-related matrix-associated actin-dependent regulator of chromatin subfamily E member 1 isoform X1 | 3.11 |
| si:ch73-167i17.6 | Regulator of G-protein signaling 9-binding protein | 2.22 |
| LOC563082 | Complexin-1 | 2.14 |
| ttc7b wu:fj46e11 zgc:153460 | Tetratricopeptide repeat protein 7B isoform X1 | 3.45 |
| dip2ca | Disco-interacting protein 2 homolog C isoform X1 | 2.95 |
| lrba | Lipopolysaccharide-responsive and beige-like anchor protein isoform X1 | 2.49 |
| wu:fb14d09 | Aldehyde dehydrogenase, mitochondrial | 1.64 |
| tpra | Nucleoprotein TPR isoform X1 | 2.56 |
| farp2 | FERM, ARHGEF and pleckstrin domain-containing protein 2 isoform X1 | 1.93 |
| si:ch211-181d7.2 | NACHT, LRR and PYD domains-containing protein 3 isoform X1 | 2.25 |
| rab3gap2 RAB3 RAB3GAP si:ch211-214p16.5 si:zc214p16.5 wu:fb94b08 zgc:158161 | Rab3 GTPase-activating protein non-catalytic subunit isoform X1 | 2.57 |
| wu:fa11g10 fa11g10 fb23g05 wu:fb23g05 zgc:86792 zgc:56095 | Ferritin | 2.86 |
| nme2a LOC137487198 | Nucleoside diphosphate kinase (EC 2.7.4.6) | 3.64 |
| afdna | Afadin isoform X6 | 2.57 |
| add1 bZ1H20.1 cb787 zgc:158352 | Alpha-adducin isoform X1 | 3.78 |
| atp2b3b | Calcium-transporting ATPase (EC 7.2.2.10) | 2.8 |
| **Gene Names** | **Protein names** | **Log2-fold change**  **(upregulated)** |
| LOC100330916 | Uncharacterized protein isoform X2 | 2.02 |
| dnm1a dnm1 si:dkey-246l19.2 | dynamin GTPase (EC 3.6.5.5) | 3.25 |
| smarcc2 | SWI/SNF complex subunit SMARCC2 (SWI/SNF-related, matrix-associated, actin-dependent regulator of chromatin, subfamily c, member 2) | 1.88 |
| pmt zgc:153034 | phosphoethanolamine N-methyltransferase (EC 2.1.1.103) | 2.04 |
| cps1 | carbamoyl-phosphate synthase (ammonia) (EC 6.3.4.16) | 1.95 |
| LOC100536500 | Cysteine-rich venom protein natrin-1 | 1.66 |
| arr3b arr3 si:bz46j2.6 zgc:73331 | Arrestin 3b, retinal (X-arrestin) isoform X1 | 2.38 |
| nedd4a | E3 ubiquitin-protein ligase NEDD4 isoform X1 | 2.67 |
| LOC101882642 | Sterile alpha motif domain-containing protein 9 | 4.83 |
| stat5a sb:eu615 Stat5 stat5 stat5.1 stat5b | Signal transducer and activator of transcription 5A isoform X1 | 1.87 |
| abcc1 | Multidrug resistance-associated protein 1 isoform X2 | 2.66 |
| clasp1a si:dkey-219c10.5 | CLIP-associating protein 1a isoform X1 | 2.9 |
| papolg zgc:63762 | polynucleotide adenylyltransferase (EC 2.7.7.19) | 1.5 |
| fn1b cb1057 fn1l fn3 wu:fa14f11 wu:fb03c02 | Fibronectin | 2.47 |
| rtf1 fb33g03 wu:fa04h07 wu:fb33g03 | RNA polymerase-associated protein RTF1 homolog | 2.26 |
| hspb1 cb153 cb660 hsp1 hsp25 hsp27 id:ibd2821 sb:cb660 zgc:103437 | Heat shock protein beta-1 (Heat shock 27 kDa protein) | 2.38 |
| vps16 im:6907749 MGC162584 wu:fc34a07 wu:fl62a06 zgc:162584 | Vacuolar protein sorting-associated protein 16 homolog | 1.62 |
| si:ch211-284e20.8 | Si:ch211-284e20.8 protein (Uncharacterized protein LOC563738 precursor) | 1.78 |
| sdr16c5a zgc:76925 | Short chain dehydrogenase/reductase family 16C, member 5a (EC 1.1.1.105) | 2.22 |
| rpz5 fd50h12 rapunzel5 wu:fd50h12 zgc:171577 | Rapunzel 5 (Zgc:171577 protein) | 2.62 |
| lrrc47 fe50f06 si:ch211-147a11.7 wu:fe50f06 | Leucine-rich repeat-containing protein 47 (Si:ch211-147a11.7 protein) | 2.37 |
| si:dkey-20d21.12 | Si:dkey-20d21.12 (Uncharacterized protein LOC556245) | 2.24 |
| rac3b zgc:175209 | Rac family small GTPase 3b (Ras-related C3 botulinum toxin substrate 3b) (Zgc:175209 protein) | 3.17 |
| pfdn5 wu:fa09c11 wu:fb38c05 zgc:100894 | Pfdn5 protein (Prefoldin 5) (Prefoldin subunit 5) | 1.92 |
| aqr zgc:63611 | RNA helicase aquarius (EC 3.6.4.13) (Zgc:63611 protein) | 2.24 |
| uroc1 zgc:194768 | urocanate hydratase (EC 4.2.1.49) (Imidazolonepropionate hydrolase) | 1.87 |
| gpc4 chunp6920 fc47a08 fe05f10 gpc4/6 gpc6 kny knypek wu:fc47a08 wu:fe05f10 zgc:194854 | Glypican-4 precursor (Knypek) | 2.39 |
| ugt1b2 Ugt1b2 | UDP-glucuronosyltransferase (EC 2.4.1.17) | 2.25 |
| aqp9b aqp9 | Aquaporin-9b | 3.95 |
| frem2a bla | FRAS1-related extracellular matrix 2a (FRAS1-related extracellular matrix protein 2a isoform X1) (Fras1-like extracellular matrix protein 2a) | 1.88 |
| ptdss2 si:ch1073-158c2 si:ch1073-279O1 | Phosphatidylserine synthase 2 (PSS-2) (PtdSer synthase 2) (EC 2.7.8.29) (Serine-exchange enzyme II) | 2.07 |
| nbeaa im:7138260 nbea si:ch211-125n11.1 zgc:113510 | Neurobeachin a | 3.32 |
| agtrap im:6894391 | Angiotensin II receptor-associated protein | 2.71 |
| abhd10b im:7144007 tagln3b | Palmitoyl-protein thioesterase ABHD10, mitochondrial (EC 3.1.1.93) (EC 3.1.2.22) (Acyl-protein thioesterase ABHD10) (Alpha/beta hydrolase domain-containing protein 10) (Mycophenolic acid acyl-glucuronide esterase, mitochondrial) | 2.69 |
| stag1b | Cohesin subunit SA (SCC3 homolog) (Stromal antigen) | 1.7 |
| si:dkey-33m11.8 | trypsin (EC 3.4.21.4) | 1.92 |
| naa25 | N-alpha-acetyltransferase 25, NatB auxiliary subunit | 3.87 |
| nlrx1 | NLR family member X1 | 1.59 |
| h6pd | GDH/6PGL endoplasmic bifunctional protein (Hexose-6-phosphate dehydrogenase (glucose 1-dehydrogenase)) | 1.42 |
| mrpl45 zgc:56480 | Large ribosomal subunit protein mL45 (39S ribosomal protein L45, mitochondrial) | 2.96 |
| fam120c | Constitutive coactivator of PPAR-gamma-like protein 2 (Family with sequence similarity 120C) | 3.06 |
| abcc2 mrp2 zgc:66072 | ABC-type glutathione-S-conjugate transporter (EC 7.6.2.3) | 2.54 |
| sltm wu:fd12b08 zgc:65985 | SAFB-like transcription modulator (SAFB-like, transcription modulator) | 2.43 |
| **Gene Names** | **Protein names** | **Log2-fold change**  **(upregulated)** |
| rbp3 irbp | Retinol-binding protein 3 | 4.88 |
| pik3ca | phosphatidylinositol-4,5-bisphosphate 3-kinase (EC 2.7.1.153) | 2.75 |
| zgc:77077 zgc:56585 | Uncharacterized protein LOC393297 isoform 1 (Zgc:56585) | 2.39 |
| slc12a4 | Solute carrier family 12 member 4 | 2.01 |
| dhrs11b.2 zgc:92630 | Dehydrogenase/reductase SDR family member 11-like (Zgc:92630) | 3.13 |
| mical3a si:dkeyp-122e2.1 | Protein-methionine sulfoxide oxidase mical3a (EC 1.14.13.225) (Molecule interacting with CasL protein 3A) (MICAL-3A) | 2.19 |
| pik3c2b | Phosphatidylinositol 4-phosphate 3-kinase C2 domain-containing subunit beta (Phosphatidylinositol-4-phosphate 3-kinase, catalytic subunit type 2 beta) | 1.82 |
| pus1 wu:fb98h11 zgc:103516 | Pseudouridine synthase 1 (tRNA pseudouridine synthase A (EC 5.4.99.12)) | 2.9 |
| hspa8b si:dkey-4p15.3 | Heat shock protein family A (Hsp70) member 8b | 3.59 |
| cul5a cul5 wu:fd17d09 wu:fi20h12 xx:11fd17d09 zgc:66185 | Cullin-5a | 3.32 |
| sec61g zgc:92840 | Protein transport protein Sec61 subunit gamma | 2.37 |
| ndufa9a ndufa9 wu:fc41f12 zgc:112513 | NADH dehydrogenase [ubiquinone] 1 alpha subcomplex subunit 9, mitochondrial (Complex I-39kD) (NADH-ubiquinone oxidoreductase 39 kDa subunit) | 2.27 |
| sgca | Alpha-sarcoglycan (Sarcoglycan, alpha) | 3.66 |
| myo10l1 | Myosin X,-like 1 (Unconventional myosin-X) | 2.13 |
| col22a1 col9a1 prp | Collagen alpha-1(XXII) chain precursor (Collagen type XXII alpha 1 chain) | 2.84 |
| faah2b zgc:153568 | Fatty-acid amide hydrolase 2-B (EC 3.5.1.99) | 2.58 |
| ssuh2 ssuh2rs1 zgc:153440 | Protein SSUH2 homolog (Protein ssu-2 homolog) | 2.4 |
| tkfc dak zgc:153296 | Triokinase/FMN cyclase (EC 2.7.1.28) (EC 2.7.1.29) (EC 4.6.1.15) (Bifunctional ATP-dependent dihydroxyacetone kinase/FAD-AMP lyase (cyclizing)) | 1.88 |
| mif MIF | Macrophage migration inhibitory factor (EC 5.3.2.1) (EC 5.3.3.12) (L-dopachrome isomerase) (L-dopachrome tautomerase) (Phenylpyruvate tautomerase) | 1.78 |
| glsa glsl | glutaminase (EC 3.5.1.2) | 1.97 |
| wu:fa96d06 fa96d06 zgc:136929 | C-terminal-binding protein 1 (Zgc:136929) | 2.94 |
| gmpr2 GMPR wu:fb63f02 zgc:136869 | GMP reductase (GMPR) (EC 1.7.1.7) (Guanosine 5'-monophosphate oxidoreductase) (Guanosine monophosphate reductase) | 2.05 |
| crp4 CRP zgc:136586 | Pentraxin family member | 2.89 |
| med24 lsn thrap4 trap100 | Mediator of RNA polymerase II transcription subunit 24 (Mediator complex subunit 24) (Protein lessen) (Thyroid hormone receptor-associated protein 4 homolog) (Trap100 homolog) | 1.48 |
| fut9d im:6895158 zgc:171333 zgc:162337 | Fucosyltransferase (EC 2.4.1.-) | 2.46 |
| ptcd3 zgc:123014 | Small ribosomal subunit protein mS39 (Pentatricopeptide repeat domain-containing protein 3, mitochondrial) | 2.43 |
| grk7a grk7-1 dkeyp-13a3.1 | Rhodopsin kinase grk7a (EC 2.7.11.14) (G protein-coupled receptor kinase 7-1) (G-protein-coupled receptor kinase 7A) | 1.99 |
| golt1bb golt1b wu:fb96a02 zgc:114204 | Golgi transport 1Bb (Zgc:114204) | 3.74 |
| gpx7 im:6902827 zgc:112293 | Glutathione peroxidase | 2.37 |
| mrps17 zgc:110031 | 28S ribosomal protein S17, mitochondrial (Mitochondrial ribosomal protein S17) (Zgc:110031) | 1.85 |
| nsdhl zgc:112474 | NAD(P) dependent steroid dehydrogenase-like (Nsdhl protein) (Sterol-4-alpha-carboxylate 3-dehydrogenase, decarboxylating (EC 1.1.1.170)) | 2.84 |
| naa40 nat11 zgc:110241 | N-alpha-acetyltransferase 40 (EC 2.3.1.257) (N-acetyltransferase 11) (N-alpha-acetyltransferase D) (NatD) | 1.84 |
| actr2b arp2b si:dkey-66m17.2 zgc:110550 | Actin-related protein 2-B (Actin-like protein 2-B) | 3.06 |
| zgc:110843 | Uncharacterized protein LOC541492 (Zgc:110843) | 1.91 |
| erlin1 si:ch211-223p8.2 zgc:110547 | Erlin-1 (Endoplasmic reticulum lipid raft-associated protein 1) | 2.44 |
| slc27a1a fc10e12 slc27a1 wu:fc10e12 zgc:101649 | long-chain-fatty-acid--CoA ligase (EC 6.2.1.3) (Long-chain-fatty-acid--CoA ligase) | 2.12 |
| wu:fd55e12 zgc:113054 | Uncharacterized protein LOC541322 (Zgc:113054) | 1.93 |
| tfr1a | Transferrin receptor protein 1 | 2.89 |
| kera | Keratocan | 3.03 |
| **Gene Names** | **Protein names** | **Log2-fold change**  **(upregulated)** |
| mcm3 cb32 chunp6867 MCM3z wu:fa26g03 | DNA replication licensing factor MCM3 (EC 3.6.4.12) | 2.77 |
| farsb cb428 farsl zgc:92055 | Phenylalanine--tRNA ligase beta subunit (EC 6.1.1.20) (Phenylalanyl-tRNA synthetase beta subunit) | 2.47 |
| cyp2ad3 cyp2j27 si:dkey-183n20.10 | Cytochrome P450, family 2, subfamily AD, polypeptide 3 (Cytochrome P450, family 2, subfamily J, polypeptide 27) | 3.09 |
| pgrmc1 fa94d03 wu:fa94d03 wu:fq25e02 zgc:103577 | Membrane-associated progesterone receptor component 1 | 1.77 |
| gcdhb gcdhl zgc:103477 | Glutaryl-CoA dehydrogenase, mitochondrial (EC 1.3.8.6) | 1.92 |
| s100a10a zgc:101880 | Protein S100 (S100 calcium-binding protein) | 1.71 |
| opcml zgc:92901 | Opioid-binding protein/cell adhesion molecule precursor (Zgc:92901) | 1.47 |
| shbg shbgl wu:fb66h07 wu:fd55b08 zgc:123063 | Sex hormone-binding globulin | 2.94 |
| slc51a osta zgc:92111 | Organic solute transporter subunit alpha (OST-alpha) (Solute carrier family 51 subunit alpha) | 2.68 |
| ccdc47 zgc:92099 | PAT complex subunit CCDC47 (Coiled-coil domain-containing protein 47) | 1.84 |
| flad1 zgc:91843 | FAD synthase (EC 2.7.7.2) (FAD pyrophosphorylase) (FMN adenylyltransferase) (Flavin adenine dinucleotide synthase) [Includes: Molybdenum cofactor biosynthesis protein-like region; FAD synthase region] | 2.3 |
| ndufv1 wu:fc01f01 wu:fc12f12 zgc:86620 | NADH dehydrogenase [ubiquinone] flavoprotein 1, mitochondrial (EC 7.1.1.2) | 2.93 |
| ralba ralb zgc:100801 | Ras-related protein Ral-B (EC 3.6.5.2) (V-ral simian leukemia viral oncogene homolog Ba (ras-related)) (Zgc:100801) | 1.58 |
| napgb cb476 napg zgc:92890 | Gamma-soluble NSF attachment protein (N-ethylmaleimide-sensitive factor attachment protein gamma) | 2.8 |
| faah2a zgc:92625 | Fatty-acid amide hydrolase 2-A (EC 3.5.1.99) | 1.78 |
| kars1 cb530 kars wu:fa16h02 zgc:92483 | Lysine--tRNA ligase (EC 6.1.1.6) (Lysyl-tRNA synthetase) | 2.7 |
| tfip11 stip zgc:86644 | Tuftelin-interacting protein 11 (Septin and tuftelin-interacting protein 1) (STIP-1) | 2.07 |
| sars1 sars | Serine--tRNA ligase, cytoplasmic (EC 6.1.1.11) (Seryl-tRNA synthetase) (SerRS) | 2.65 |
| hprt1l zgc:86643 | Hypoxanthine phosphoribosyltransferase (EC 2.4.2.8) | 2.87 |
| cops2 csn2 zgc:86624 | COP9 signalosome complex subunit 2 (Signalosome subunit 2) | 2.33 |
| lta4h zgc:85809 | Leukotriene A(4) hydrolase (LTA-4 hydrolase) (EC 3.3.2.6) | 2.68 |
| ilvbl hacl2 zgc:66376 zgc:85697 | 2-hydroxyacyl-CoA lyase 2 (EC 4.1.2.-) (Acetolactate synthase-like protein) (IlvB-like protein) | 3.57 |
| mccc2 si:dkey-57m14.1 zgc:85685 | methylcrotonoyl-CoA carboxylase (EC 6.4.1.4) (3-methylcrotonyl-CoA carboxylase 2) (3-methylcrotonyl-CoA carboxylase non-biotin-containing subunit) (3-methylcrotonyl-CoA:carbon dioxide ligase subunit beta) | 2.01 |
| st13 wu:fd15g02 zgc:73267 | Hsc70-interacting protein (ST13 Hsp70-interacting protein) (Suppression of tumorigenicity 13 (Colon carcinoma) (Hsp70 interacting protein)) | 4.42 |
| cacybp zgc:76993 | Calcyclin-binding protein | 2.75 |
| fam49bb si:dkeyp-105g12.2 zgc:76981 | Family with sequence similarity 49 member Bb (Uncharacterized protein LOC404610) (Zgc:76981) | 2.88 |
| adrm1b | Proteasomal ubiquitin receptor ADRM1 | 2.74 |
| emc6 tmem93 zgc:77320 | ER membrane protein complex subunit 6 (Transmembrane protein 93) | 3.16 |
| rpl19 zgc:77733 | Large ribosomal subunit protein eL19 (60S ribosomal protein L19) | 3.83 |
| arglu1a si:ch211-149b20.2 zgc:55375 | Arginine and glutamate-rich protein 1-A | 3.24 |
| gnao1b zgc:73153 | Guanine nucleotide-binding protein G(o) subunit alpha | 1.99 |
| ddx23 wu:fi39b12 zgc:63742 | RNA helicase (EC 3.6.4.13) | 2.74 |
| calb2b calb2 wu:fq17g09 zgc:73099 | Calbindin 2, (Calretinin) (Calbindin 2b) | 4.52 |
| ddb1 zgc:63840 | DNA damage-binding protein 1 (Zgc:63840) | 1.41 |
| hsc70 zgc:63663 | Heat shock cognate 70 (Uncharacterized protein LOC393586) (Zgc:63663) | 2.15 |
| cisd1 si:dkey-18n13.4 zgc:63561 | CDGSH iron sulfur domain 1 (CDGSH iron-sulfur domain-containing protein 1 (EC 2.6.1.3)) (Zgc:63561) | 2.03 |
| eif3d eif3s7 si:dkey-165I8.6 | Eukaryotic translation initiation factor 3 subunit D (eIF3d) (Eukaryotic translation initiation factor 3 subunit 7) | 4.22 |
| gnai2a GNAI2 Gnai2 gnai2l wu:fb10b04 wu:fb19c04 zgc:92609 | Guanine nucleotide-binding protein G(i) subunit alpha-2 (Adenylate cyclase-inhibiting G alpha protein) | 3.73 |
| **Gene Names** | **Protein names** | **Log2-fold change**  **(upregulated)** |
| h2az2a | Histone H2A.V (H2A.F/Z) | 3.67 |
| pklr wu:fd15e01 wu:fi37e08 | Pyruvate kinase (EC 2.7.1.40) | 3.61 |
| mtpn | Myotrophin | 3.17 |
| ptk2bb im:6900512 ptk2b ptk2bl pyk2 | non-specific protein-tyrosine kinase (EC 2.7.10.2) | 1.95 |
| cisd2 dkey-162b23.1 zgc:64148 | CDGSH iron-sulfur domain-containing protein 2 | 2.47 |
| abhd14b zgc:64031 | Putative protein-lysine deacylase ABHD14B (Alpha/beta hydrolase domain-containing protein 14B) | 1.99 |
| ivd sb:cb425 si:dz181p14.1 wu:fb34d07 | Isovaleryl-CoA dehydrogenase, mitochondrial (EC 1.3.8.1) (EC 1.3.8.4) (Butyryl-CoA dehydrogenase) | 2.8 |
| cyp3c1 CYP3A wu:fa96d12 wu:fc26b04 zgc:56626 | Cytochrome P450, family 3, subfamily c, polypeptide 1 | 3.61 |
| mgst3a mgst3 zgc:56518 | Microsomal glutathione S-transferase 3a (EC 2.5.1.18) (Zgc:56518) | 2.41 |
| ppp2r5eb ppp2r5e ppp2r5e1 wdb2 | Serine/threonine protein phosphatase 2A regulatory subunit | 3.38 |
| tbca zgc:56262 | Tubulin-specific chaperone A | 2.33 |
| ephx1 zgc:56126 | Epoxide hydrolase (EC 3.3.2.9) | 3.5 |
| pdlim5b zgc:56116 zgc:86712 | PDZ and LIM domain protein 5b isoform 2 (Zgc:56116) | 2.08 |
| sirt2 zgc:77003 | NAD-dependent protein deacetylase sirtuin-2 (EC 2.3.1.286) (NAD-dependent protein defatty-acylase sirtuin-2) (EC 2.3.1.-) (Regulatory protein SIR2 homolog 2) (SIR2-like protein 2) | 1.43 |
| sephs1 si:ch211-220f12.5 | Selenide, water dikinase 1 (EC 2.7.9.3) (Selenium donor protein 1) (Selenophosphate synthase 1) | 2.1 |
| papss2b id:ibd2761 Papss1 papss2 sb:cb868 wu:fb12e05 zgc:55851 zgc:85655 | 3'-phosphoadenosine 5'-phosphosulfate synthase 2 (Bifunctional 3'-phosphoadenosine 5'-phosphosulfate synthase 2b (EC 2.7.7.4)) | 2.47 |
| usp14 wu:fk63d09 zgc:55949 | Ubiquitin carboxyl-terminal hydrolase (EC 3.4.19.12) | 2.01 |
| ankmy2a fi46e08 wu:fi46e08 zgc:55491 | Ankyrin repeat and MYND domain-containing protein 2a (Zgc:55491) | 3.4 |
| sh3gl2a sh3gl2 zgc:55616 | SH3 domain-containing GRB2-like 2a, endophilin A1 (SH3-domain GRB2-like 2) (Sh3gl2 protein) | 2.28 |
| lamb4 | Laminin subunit beta-4 | 2.19 |
| cldn1 cldn19 | Claudin | 1.89 |
| casp3a casp3 zgc:100890 | Caspase-3 (EC 3.4.22.56) | 2.45 |
| map2k6 map2k3 | Dual specificity mitogen-activated protein kinase kinase 6 (MAP kinase kinase 6) (MAPKK 6) (EC 2.7.12.2) (Mitogen-activated protein kinase kinase 3) (zMKK3) | 3.16 |
| copz1 CHUNP6876 | Coatomer subunit zeta | 1.81 |
| cat | Catalase (EC 1.11.1.6) | 3.32 |
| pcna | Proliferating cell nuclear antigen (PCNA) | 2.1 |
| gsk3ab GSK-3[a] GSK-3[b] GSK3 gsk3a | [tau protein] kinase (EC 2.7.11.26) | 2.26 |

**Table S2.** DRPs identified using zebrafish protein database (downregulated proteins).

| **Gene Names** | **Protein names** | **Log2-fold change**  **(downregulated)** |
| --- | --- | --- |
| ocrl wu:fi09g03 zgc:152864 | phosphoinositide 5-phosphatase (EC 3.1.3.36) | 3.24 |
| utp20 | Small subunit processome component 20 homolog (UTP20 small subunit processome component) | 1.51 |
| atp2a3 | Calcium-transporting ATPase (EC 7.2.2.10) | 1.42 |
| esyt1b | Extended synaptotagmin-1 isoform X1 | 2.5 |
| zgc:66156 | Myosin heavy chain, fast skeletal muscle | 3.78 |
| si:dkey-238c7.16 fc76h06 wu:fc76h06 DKEY-238C7.16-001 | Novel protein (Si:dkey-238c7.16) (Uncharacterized protein LOC559078) | 2.58 |
| si:dkey-9i23.15 | Si:dkey-9i23.15 (Transmembrane protein 109 precursor) | 2.52 |
| ngef | Ephexin-1 isoform X1 (Neuronal guanine nucleotide exchange factor) | 6.23 |
| zc3h4 | Zinc finger CCCH domain-containing protein 4 (Zinc finger CCCH-type-containing 4) | 2.77 |
| ap5b1 si:ch211-146f2.3 | AP-5 complex subunit beta-1 (Adaptor-related protein complex 5 beta subunit) | 6.82 |
| rint1 si:dkey-202b22.4 zgc:153458 | RAD50-interacting protein 1 (Si:dkey-202b22.4) | 4.31 |
| pmpcb zgc:110738 | Mitochondrial-processing peptidase subunit beta (EC 3.4.24.64) | 1.67 |
| acat2 fb10a06 fb53f08 wu:fb10a06 wu:fb53f08 | Acat2 protein (Acetyl-CoA acetyltransferase 2) (Acetyl-CoA acetyltransferase, cytosolic (EC 2.3.1.9)) | 2.17 |
| thap12a fb55g11 prkrir prkrira prkrirl wu:fb55g11 zgc:55697 | Prkrir protein (Protein-kinase, interferon-inducible double stranded RNA dependent inhibitor) | 2.02 |
| cpsf4 nar | Cleavage and polyadenylation specificity factor subunit 4 (CPSF 30 kDa subunit) | 1.36 |

**Table S3.** DRPs identified using human protein database (downregulated proteins).

| **Gene names** | **Protein names** | **Log2-fold change**  **(downregulated)** |
| --- | --- | --- |
| DDX39A | ATP-dependent RNA helicase | 4.53 |
| CLUH | Clustered mitochondria protein homolog | 4.12 |
| HSPB1 | Heat shock protein beta-1 | 5.07 |
| MYL2 | Myosin regulatory light chain 2 | 3.32 |
| MATN1 | Cartilage matrix protein | 2.68 |
| PHB1 | Prohibitin 1 | 4.13 |
| RPL30 | Ribosomal protein L30 | 3.76 |
| DRAP1 | Dr1-associated corepressor | 4.68 |
| DHRS11 | Dehydrogenase/reductase SDR family member 11 | 3.33 |
